# Comprehensive evaluation of the efficacy and safety of different vitamin D combination regimens based on indirect comparisons for children with rickets: a network meta-analysis

**DOI:** 10.3389/fnut.2026.1785775

**Published:** 2026-04-08

**Authors:** Yunfeng Cao, Yan Zheng, Yaling Chen, Qihua Chen, Xinbin Xia, Ziyi Yuan, Honghui Li

**Affiliations:** 1 Hunan University of Chinese Medicine, Changsha, China; 2 The First Hospital of Hunan University of Chinese Medicine, Changsha, China

**Keywords:** children, network meta-analysis, randomized controlled trials, rickets, vitamin D

## Abstract

**Background:**

Nutritional rickets is a major pediatric bone disorder closely associated with vitamin D deficiency. Clinical practice has gradually shifted from vitamin D monotherapy toward combination regimens aiming to enhance therapeutic efficacy through complementary mechanisms. However, head-to-head evidence comparing different vitamin D-based combinations remains limited, and their relative efficacy and safety rankings are unclear.

**Objective:**

To systematically compare the efficacy and safety of different oral vitamin D–based combination therapies for nutritional rickets in children and to provide evidence-based guidance for clinical decision-making.

**Methods:**

We conducted a systematic search of PubMed, Embase, the Cochrane Library, Web of Science, CNKI, Wanfang, and VIP databases, followed by a Bayesian network meta-analysis. The study protocol was prospectively registered in PROSPERO (CRD420251029274).

**Results:**

Ten randomized controlled trials involving 867 children were included, comparing nine vitamin D–based combination regimens with vitamin D3 monotherapy. Pairwise meta-analyses showed that combination therapies significantly improved serum 25-hydroxyvitamin D levels, reduced bone-specific alkaline phosphatase, and increased serum calcium and phosphate concentrations, without an increased risk of adverse events. Subgroup analyses indicated significantly greater benefits among term infants than preterm infants (*P* < 0.001). Network meta-analysis revealed distinct relative advantages across outcomes among different combinations. Sensitivity analyses confirmed the robustness of the findings.

**Conclusion:**

Vitamin D-based combination therapies are overall more effective than vitamin D3 monotherapy in improving biochemical markers of bone metabolism in children with rickets, without compromising safety. Different regimens exhibit outcome-specific advantages, with clearer benefits observed in term infants. These findings support individualized, goal-oriented combination strategies, although further high-quality trials are required to strengthen the evidence base.

**Systematic review registration:**

https://www.crd.york.ac.uk/prospero/, identifier CRD420251029274.

## 1 Introduction

Rickets is a pediatric metabolic bone disorder primarily characterized by defective mineralization of the bone matrix (1). Its pathophysiology is most commonly driven by vitamin D deficiency or dysregulated vitamin D metabolism, resulting in disturbances of calcium–phosphorus homeostasis. These abnormalities lead to reduced intestinal calcium absorption and impaired renal tubular phosphate reabsorption, ultimately causing hypophosphatemia and defective skeletal mineralization (2). Hallmark pathological features include disruption of growth plate architecture, excessive osteoid accumulation, and skeletal softening with deformity. Clinically, affected children often present with delayed skeletal development, lower limb deformities (such as genu varum or genu valgum), thoracic abnormalities, and delayed motor development (1, 3–5). Without timely intervention, rickets can exert long-lasting adverse effects on somatic growth, physical function, and overall quality of life. Epidemiological evidence indicates that rickets remains highly prevalent in low- and middle-income regions, including parts of Asia, the Middle East, and Africa, with reported prevalence rates ranging from approximately 1% to 24% (6). Notably, an increasing incidence has also been observed in several high-income countries, such as the United States, the United Kingdom, Australia, and Denmark. Consequently, the effective prevention, early intervention, and systematic management of pediatric rickets have emerged as an urgent global public health priority.

Vitamin D constitutes the cornerstone of both prevention and treatment of rickets by enhancing intestinal calcium and phosphate absorption and regulating bone metabolism (7). However, accumulating evidence suggests that vitamin D monotherapy has inherent limitations. A meta-analysis encompassing 32 randomized controlled trials (RCTs) demonstrated that (8), even with high-dose vitamin D supplementation, improvements in biochemical markers of bone metabolism and radiographic outcomes remained suboptimal in a subset of patients, with only modest clinical symptom relief (9, 10).

Consequently, clinical practice has progressively shifted toward combination strategies centered on vitamin D, including co-administration with calcium or zinc supplements, probiotics, traditional herbal formulations, or other adjunctive interventions. These multimodal regimens may exert synergistic effects through multiple biological pathways, concurrently improving calcium-phosphate homeostasis, enhancing immune regulation, and optimizing intestinal function. Such integrated approaches are therefore more likely to promote skeletal health and overall recovery, ultimately leading to improved treatment responsiveness and clinical outcomes (11, 12). In parallel, a growing body of clinical research has investigated various vitamin D-based combination therapies for rickets, with prior randomized controlled trials and conventional meta-analyses providing preliminary evidence that combined interventions may confer advantages over vitamin D monotherapy.

Nevertheless, important limitations persist in the existing literature. Most available studies focus on comparisons between a single combination regimen and vitamin D monotherapy, while direct head-to-head comparisons among different combination strategies are largely lacking. In addition, heterogeneity in outcome definitions and safety assessments across studies has resulted in fragmented evidence, thereby constraining its clinical applicability. Network meta-analysis (NMA) offers a robust methodological framework to integrate both direct and indirect evidence, enabling simultaneous comparison and ranking of multiple interventions. Accordingly, applying this approach to systematically evaluate the efficacy and safety of diverse vitamin D-based combination therapies in pediatric rickets is both methodologically justified and clinically relevant, providing a more comprehensive evidence base to inform therapeutic decision-making.

## 2 Method

This study was conducted in strict accordance with the PRISMA-NMA reporting guidelines for systematic reviews and network meta-analyses. The completed PRISMA checklist is provided in Supplementary material 1. The study protocol has been prospectively registered with PROSPERO (registration number: CRD420251029274) and is currently under review. As this work constitutes a secondary analysis of data derived exclusively from previously published randomized controlled trials, no additional ethical approval or informed consent was required.

### 2.1 Search strategy

A comprehensive literature search was conducted across seven electronic databases, including China National Knowledge Infrastructure (CNKI), Wanfang Database, VIP Journal Integration Platform (VIP), PubMed, Cochrane Library, Embase, and Web of Science. The search covered the period from database inception to 15 December 2025, which was defined as the publication cut-off date for this review. Studies published online ahead of print before this date were also considered, whereas no prospective searches were conducted beyond the cut-off. The search strategy was developed using a combination of Medical Subject Headings (MeSH) terms and free-text keywords. Detailed search strategies for each database are provided in Supplementary material 2. To minimize potential language-related information bias, no language restrictions were applied during the initial screening; however, only studies published in Chinese or English were ultimately included in the analysis.

### 2.2 Eligibility criteria

#### 2.2.1 Eligibility criteria

Study selection was guided by the PICO framework. Eligible studies met the following criteria: (1) Study design: RCTs comparing different vitamin D–based combination therapies for the treatment of pediatric rickets; (2) Participants: Children aged 3 months to 8 years with a confirmed diagnosis of vitamin D deficiency rickets; (3) Interventions: oral vitamin D administered in combination with other pharmacological agents; (4) Comparators: placebo or oral vitamin D monotherapy, with the control intervention restricted to a single agent; and (5) Outcomes: serum 25-Hydroxyvitamin D_3_ (25-(OH)D_3_), serum bone-specific alkaline phosphatase (BALP), serum calcium, and serum phosphorus. The incidence of adverse events was also systematically recorded.

#### 2.2.2 Exclusion criteria

Studies meeting any of the following criteria were excluded: (1) incomplete data or data unsuitable for quantitative synthesis; (2) unavailability of the full text; (3) publications in languages other than Chinese or English; (4) duplicate records across databases; (5) interventions involving non-oral routes of administration (e.g., intramuscular injection); and (6) non-randomized controlled trial designs, including comparative studies, case reports, study protocols, reviews, conference abstracts, or clinical guidelines.

### 2.3 Study selection and data extraction

Study selection and data extraction were performed independently by two reviewers. First, all retrieved records were imported into EndNote X9 for deduplication. Titles and abstracts were then screened to exclude studies that were clearly irrelevant. Articles deemed potentially eligible proceeded to full-text review, during which the same two reviewers independently assessed eligibility against the predefined inclusion criteria. Data extracted included the first author, year of publication, sample size, age and sex distribution of participants, intervention and control regimens, duration of treatment, and reported outcome measures. Any discrepancies arising during the screening or data extraction process were resolved through discussion; if consensus could not be reached, a third reviewer adjudicated the final decision.

### 2.4 Risk of bias and evidence quality assessment

The risk of bias of the included studies was assessed using the Cochrane Risk of Bias tool, version 2.0 (RoB 2.0). In accordance with the Cochrane Handbook for Systematic Reviews of Interventions (version 5.1.0), methodological quality was independently evaluated by two reviewers. Any discrepancies were resolved by consensus, with consultation of a third reviewer when necessary. The assessment focused on five domains: bias arising from the randomization process, bias due to deviations from intended interventions, bias related to missing outcome data, bias in outcome measurement, and bias in the selection of the reported results.

### 2.5 Statistical analysis

Bayesian NMA was conducted using R (version 4.3.2) with the gemtc package. Model estimation was performed under a Markov chain Monte Carlo (MCMC) framework, with 2,000 burn-in iterations followed by 50,000 sampling iterations. Model convergence was assessed using trace plots, posterior density plots, and Brooks–Gelman–Rubin (BGR) diagnostics. For effect size estimation, mean difference (MD) was used for continuous outcomes and risk ratio (RR) for dichotomous outcomes, with results reported as 95% credible intervals (CIs). Statistical heterogeneity was evaluated using the *I*^2^ statistic: a fixed-effects model was applied when *I*^2^ <50% and *P* > 0.10, whereas a random-effects model was used when *I*^2^ ≥ 50% or *P* ≤ 0.10. In the presence of substantial heterogeneity, subgroup and sensitivity analyses were conducted to explore potential sources. Leave-one-out sensitivity analyses were further performed to assess the robustness of the findings and to identify influential studies. As fewer than 10 studies were available for each outcome, funnel plots were not generated to assess publication bias. Finally, treatments were ranked according to the surface under the cumulative ranking curve (SUCRA) and the probability of being the best intervention, and pairwise ranking summaries were derived accordingly.

## 3 Result

### 3.1 Result selection

**Figure 1:**
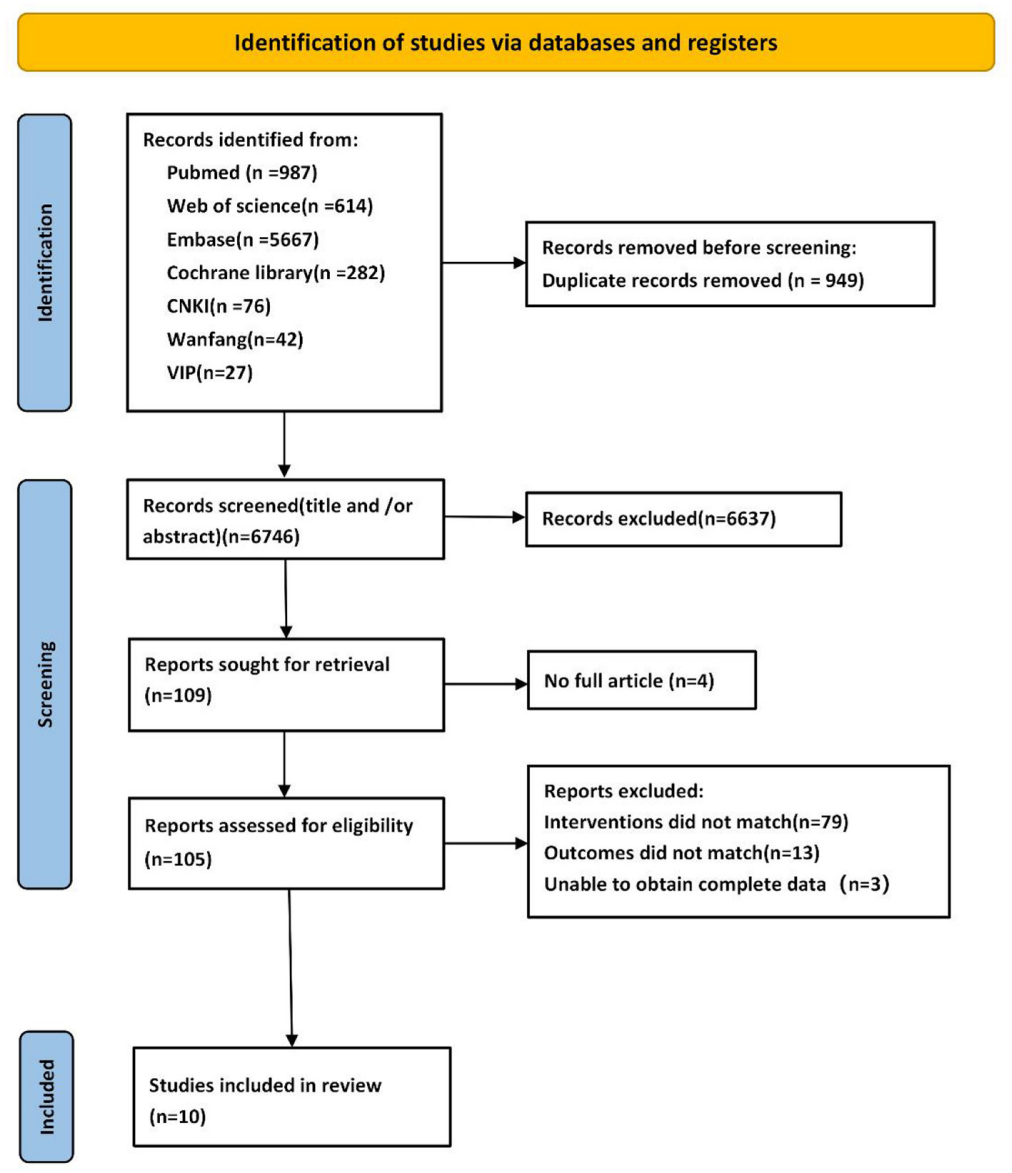
PRISMA flow diagram of literature search and study selection.

A total of 7,695 records were identified through database searching, including 76 from CNKI, 42 from Wanfang Data, 27 from VIP, 987 from PubMed, 614 from Web of Science, 5,667 from Embase, and 282 from the Cochrane Library. After deduplication using EndNote (version 2025), 949 records were removed. Title and abstract screening excluded 6,637 records, leaving 113 articles for full-text assessment of eligibility. Ultimately, 10 randomized controlled trials met the inclusion criteria and were included in the final analysis. The detailed study selection process is illustrated in Figure 1.

### 3.2 Research characteristic

A total of 10 RCTs, encompassing 867 pediatric patients with rickets, were included in this study. All participants had a confirmed clinical diagnosis of rickets. According to the intervention strategies, patients were allocated as follows: 437 children received vitamin D_3_ drops alone; 49 received vitamin D_3_ combined with Calcium and Zinc Gluconates; 50 received vitamin D_3_ plus Sijunzi decoction; 41 received vitamin D_3_ combined with pediatric multivitamin calcium granules; 32 received vitamin D_3_ combined with vitamin D-phosphate-calcium tablets; 84 received vitamin D_3_ combined with calcitriol; 34 received vitamin D_3_ combined with Xinjia Yupingfeng decoction; 30 received vitamin D_3_ combined with Saccharomyces boulardii powder; 81 received vitamin D_3_ combined with alfacalcidol; and 29 received vitamin D_3_ combined with calcium carbonate-vitamin D_3_ granules. Regarding outcome measures, seven studies reported serum 25-(OH)D_3_ levels, seven assessed BALP, six reported serum calcium and phosphorus concentrations, and an additional six studies evaluated the incidence of adverse events associated with different interventions. The baseline characteristics of the included studies are summarized in Table 1.

**Table 1:** Characteristics of included studies.

References	Age (C/I)	Gender (male/female)	Sample size (C/I)	Control group	Intervention group	Treatment course	Outcome
Jiang et al. (19)	C: (6.36 ± 1.97) month I: (6.56 ± 1.81) month	C: 29/20 I: 27/22	49/49	Vitamin D_3_: 400IU, bid	Vitamin D_3_ + Ca/Zn-Glu: 10 ml, qd	6 months	①②③
Zhang and Gao (17)	C: (1.73 ± 0.31) year I: (1.68 ± 0.26) year	C: 30/26 I: 26/24	56/50	Vitamin D_3_: 800IU, qd	Vitamin D_3_+ SJZ-T: 150 ml, bid	6 months	②⑤
Xu (16)	C: (11.86 ± 4.81) month I: (11.98 ± 4.72) month	C: 22/19 I: 23/18	41/41	Vitamin D_3_: 800IU, qd	Vitamin D_3_ + SWPG-G: 3 g, tid	3 months	①③④⑤
Huang et al. (13)	C: (2.05 ± 0.0.21) year I: (2.11 ± 0.0.19) year	C: 15/17 I: 13/19	32/32	Vitamin D_3_: 400IU, qd	Vitamin D_3_ + D_2_-CaP/Gluc: 1–2 tabs, bid -tid	2 months	①②③④
Zhang (20)	C: (2.56 ± 0.25) year I: (2.59 ± 0.33) year	C: 20/25 I: 22/23	45/45	Vitamin D_3_: 800IU, qd	Vitamin D_3_ + CAL: 0.25 μg, qd	6 months	②③④
Wang et al. (14)	C: (11.9 ± 1.04) month I: (11.1 ± 1.02) month	C: 17/16 I: 18/16	33/34	Vitamin D_3_: 2000IU, qd	Vitamin D_3_ + XJYPF-T: 30–80 ml, bid	1 months	①
Liu (15)	C: (1.93 ± 0.61) year I: (1.97 ± 0.55) year	C: 17/13 I: 15/15	30/30	Vitamin D_3_: 400–800IU, qd	Vitamin D_3_ + S-boulardii: 250 mg, qd	3 months	①②③④⑤
Hu (21)	C: (1.45 ± 0.66) month I: (1.46 ± 0.64) month	C: 39/42 I: 40/41	81/81	Vitamin D_3_: 400IU, qd	Vitamin D_3_ + Alfa: 0.1 μg/kg, qd	1 months	①②⑤
Meng and Jiao (18)	C: (13.14 ± 1.52) month I: (13.65 ± 1.77) month	C: 17/14 I: 15/14	31/29	Vitamin D_3_: 400IU, qd	Vitamin D_3_ + CaCO_3_-D_3_-G: 1 packet granules, bid	1.5 months	①③④⑤
Liu (22)	C: (2.26 ± 1.09) year I: (2.34 ± 1.13) year	C: 20/19 I: 21/18	39/39	Vitamin D_3_: 2000IU, qd	Vitamin D_3_ + CAL: 0.25 μg, qd	2 months	②③④

Ca/Zn-Glu: Calcium and Zinc Gluconates; SJZ-T: Sijunzi decoction; SWPG-G: Siwei Pugai Granules; D_2_-CaP/Gluc: Vitamin D-phosphate-calcium tablets; CAL: Calcitriol; XJYPF-T: Xinjia Yupingfeng decoction; S-boulardii: Saccharomyces boulardii; Alfa: Alfacalcidol; CaCO_3_-D_3_-G: Calcium carbonate-vitamin D3 granules. ①25-(OH)D_3_; ②BALP; ③Serum calcium; ④Serum phosphorus; ⑤Adverse actions.

### 3.3 Risk of bias of included studies

**Figure 2:**
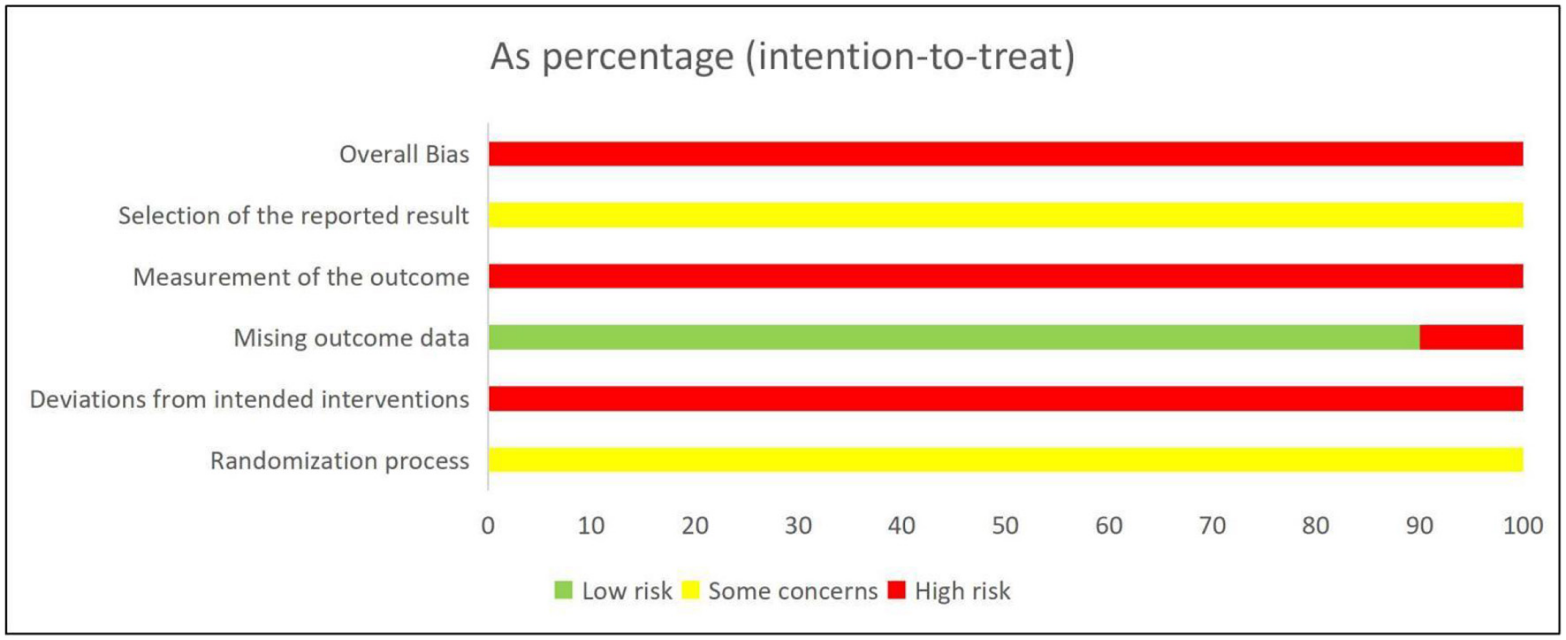
Results of the risk of bias assessment for the included studies.

With respect to bias arising from the randomization process, nine studies (13–21) reported explicit methods for random sequence generation. Specifically, six studies (14–16, 19–21) employed a random number table, three studies (13, 17, 18) allocated participants according to treatment modalities, and one study (22) merely stated that “random grouping” was used without providing methodological details. Regarding bias in outcome measurement, only one (14) study reported incomplete outcome data. In this study, two participants in the control group were excluded due to poor treatment adherence and one withdrew because of concomitant capillary bronchitis; in the intervention group, three participants were excluded owing to nonadherence and one withdrew due to viral enteritis. Overall, the methodological quality of the majority of the included studies was judged to be low. The risk of bias for each included study is presented in Figure 2.

### 3.4 Pairwise meta-analysis results

**Figure 3:**
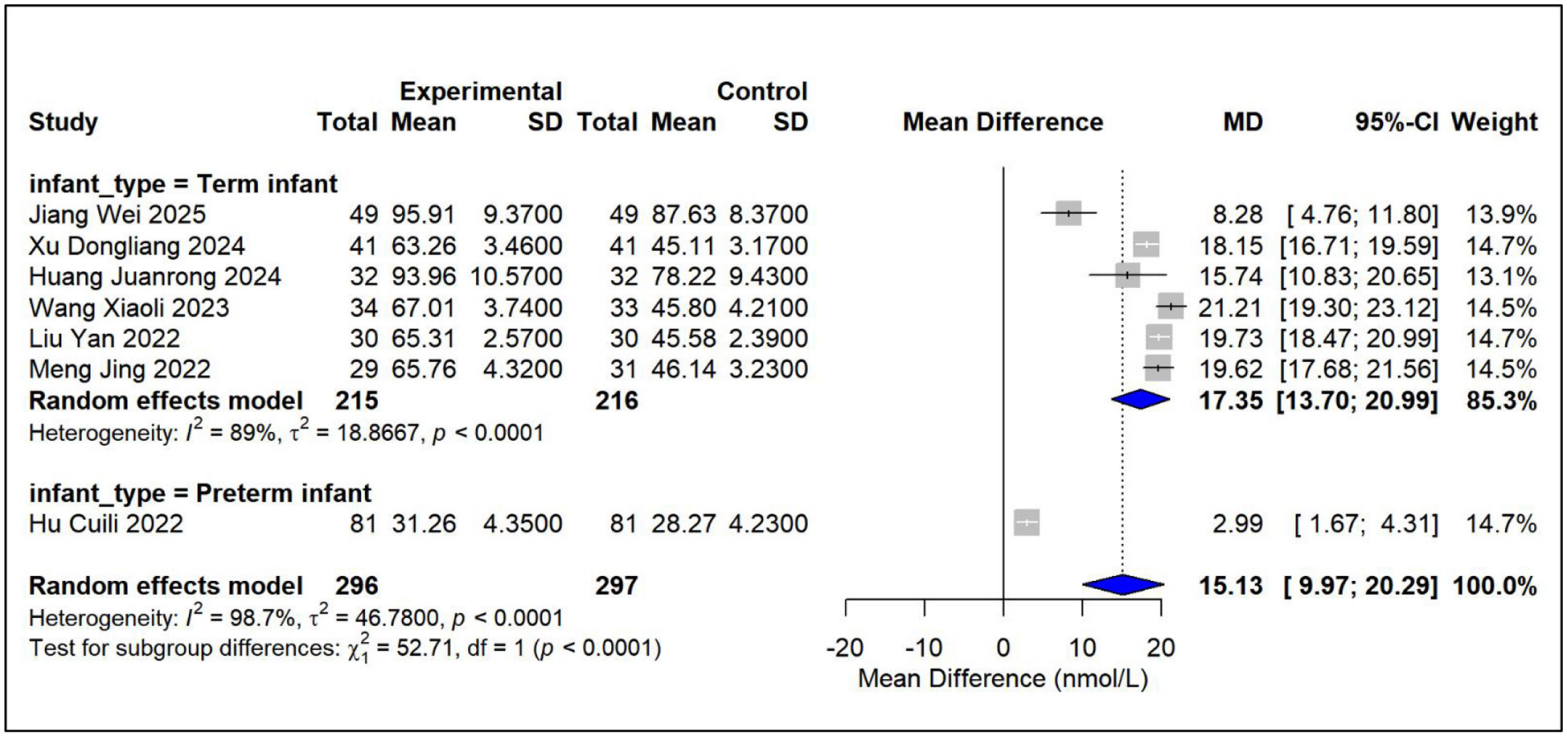
Relative effect forest plot of 25-(OH)D_3_.

**Figure 4:**
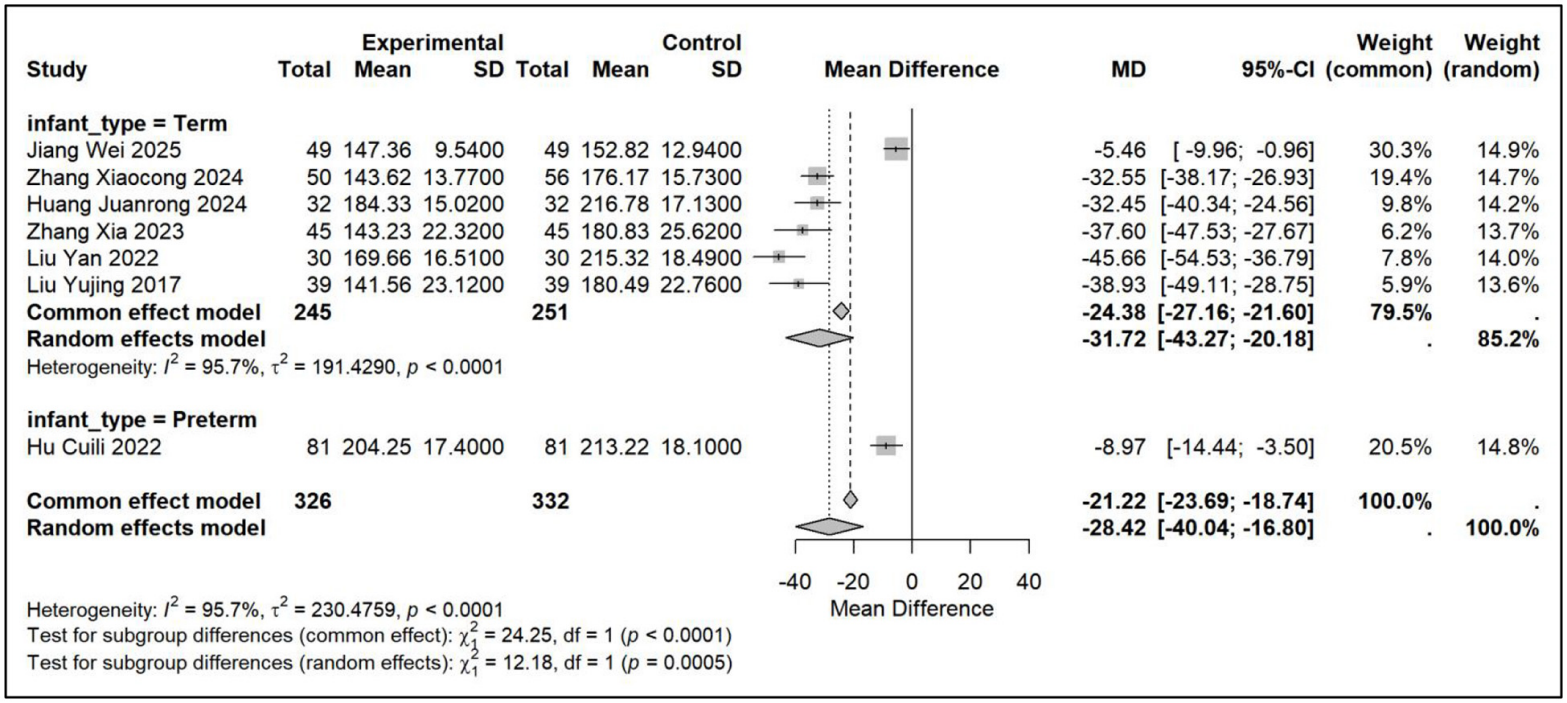
Relative effect forest plot of BALP.

**Figure 5:**
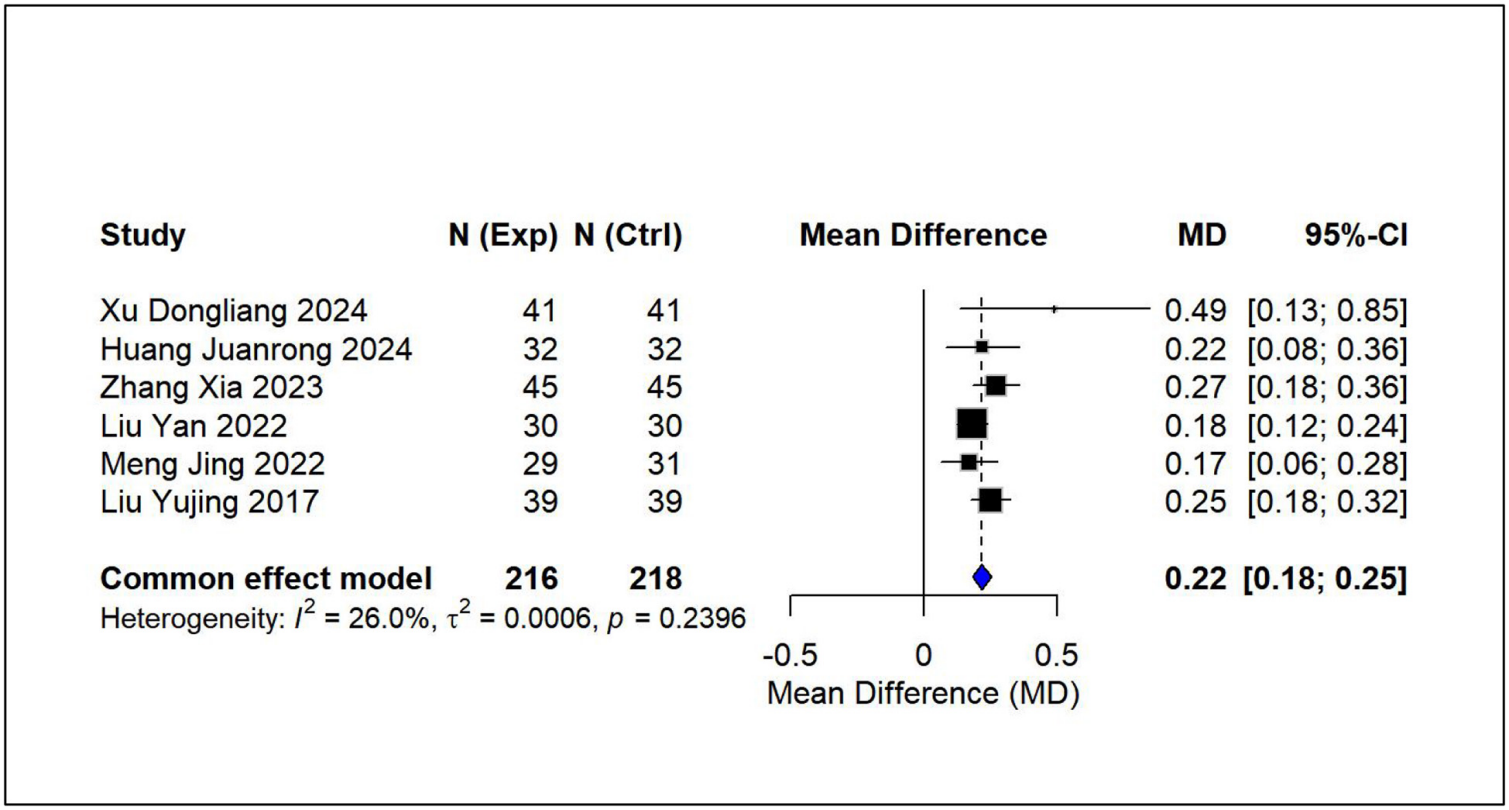
Relative effect forest plot of serum calcium.

**Figure 6:**
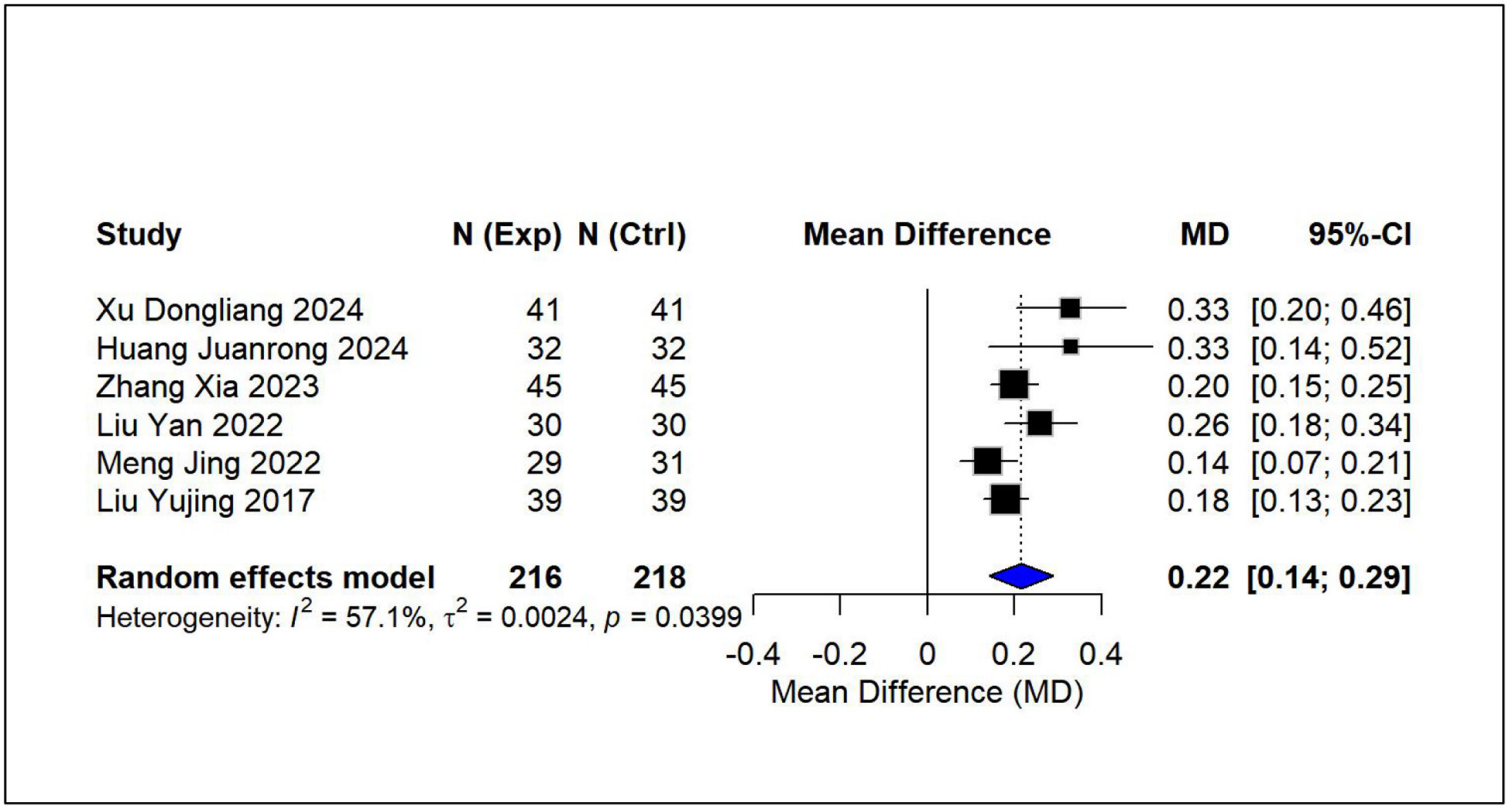
Relative effect forest plot of serum phosphorus.

**Figure 7:**
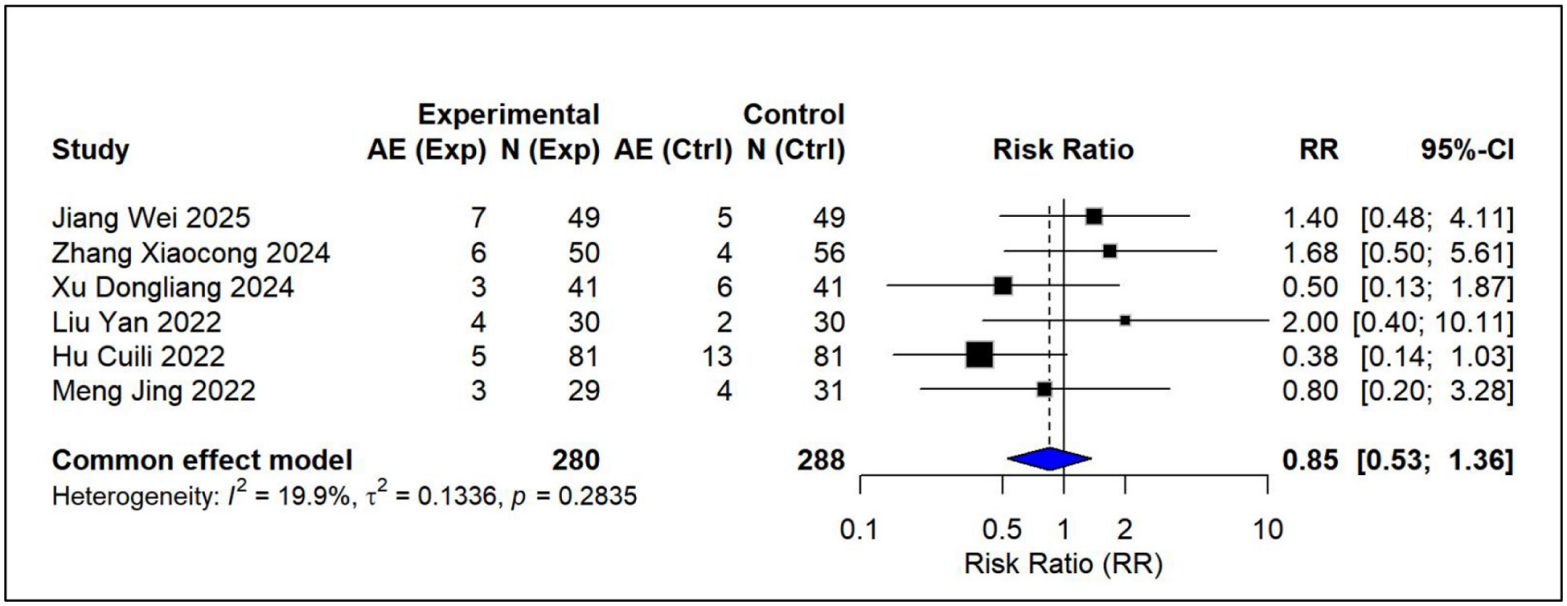
Relative effect forest plot of adverse reaction.

Using pairwise meta-analysis, we systematically evaluated the efficacy and safety of vitamin D-based combination therapies compared with vitamin D_3_ monotherapy across five key outcome measures Figures 3–7. The primary efficacy analysis demonstrated that combination therapies were overall superior to monotherapy in improving bone metabolism-related outcomes. Serum 25-(OH)D3 levels increased significantly (MD = 15.13, 95% CI [8.74, 21.52]); however, substantial between-study heterogeneity was observed (*I*^2^ = 98.7%), and therefore a random-effects model was applied (Figure 3). Levels of BALP were significantly reduced (MD = −28.42, 95% CI [−42.81, −14.03]), with similarly high heterogeneity (*I*^2^ = 95.7%), warranting the use of a random-effects model (Figure 4). Significant improvements were also observed in serum calcium (MD = 0.22 mmol/L, 95% CI [0.18, 0.25]; *I*^2^ = 26.0%) and serum phosphorus levels (MD = 0.22 mmol/L, 95% CI [0.14, 0.29]; *I*^2^ = 57.1%). Accordingly, fixed-effects and random-effects models were applied, respectively (Figures 5, 6). Safety analyses indicated no significant difference in the incidence of adverse events between the two groups (RR = 0.85, 95% CI [0.53, 1.36]), with low heterogeneity (*I*^2^ = 19.9%); thus, a fixed-effects model was used (Figure 7).

Subgroup analyses stratified by infant type (full-term vs. preterm) identified population heterogeneity as a critical determinant of treatment efficacy. (1) Significant population-level heterogeneity in treatment effects. With respect to increases in serum 25-(OH)D_3_ levels, full-term infants exhibited a substantially greater mean improvement (17.35 nmol/L) than preterm infants (2.99 nmol/L), with the between-subgroup difference reaching high statistical significance (*P* < 0.0001). Similarly, reductions in bone-specific alkaline phosphatase (BALP) levels were more pronounced among full-term infants (−31.72 U/L) than among preterm infants (−8.97 U/L), with a statistically significant subgroup difference (*P* = 0.0005). (2) Consistent findings indicate infant type as a key effect modifier. Subgroup analyses of both outcome measures demonstrated a consistent pattern, indicating that full-term infants derived markedly greater benefit from vitamin D-based combination therapies than preterm infants. Although the preterm subgroup comprised only a single study—thereby limiting the reliability and generalizability of its estimates—a clear divergence in the direction of effect was nonetheless observed. (3) Within-subgroup heterogeneity reflects variability in treatment regimens. Considerable heterogeneity persisted within the full-term subgroup (25-(OH)D_3_: *I*^2^ = 89%; BALP: *I*^2^ = 95.7%), suggesting that even within a homogeneous infant population, variations in intervention composition, dosage, treatment duration, and baseline characteristics may substantially influence therapeutic outcomes.

### 3.5 Results of network meta-analysis

**Figure 8:**
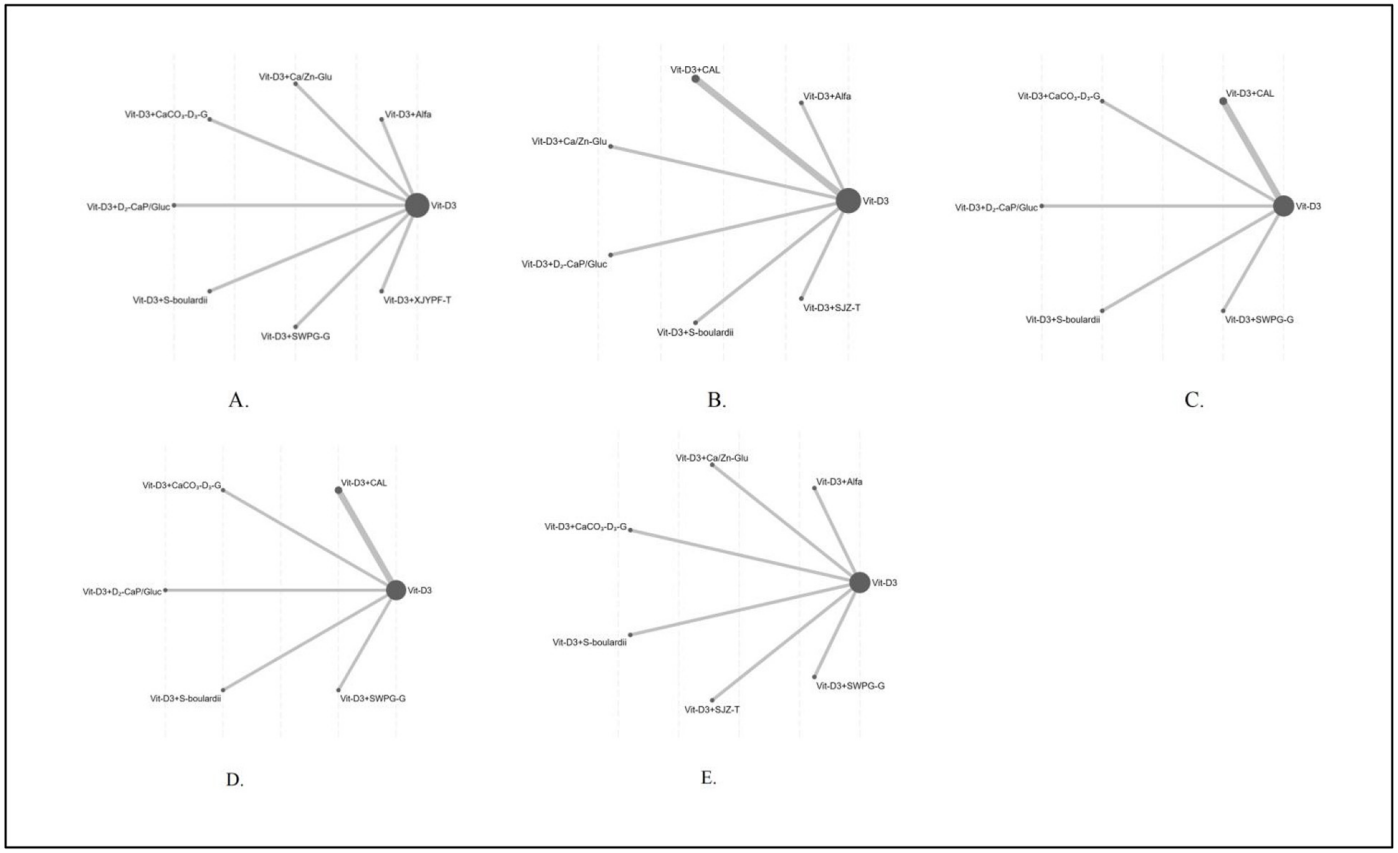
Network geometry of all interventions for outcome measures. **(A)** 25-(OH)D_3_; **(B)** BALP; **(C)** Serum calcium; **(D)** Serum phosphorus; **(E)** Adverse reaction.

The network evidence plot (Figure 8) provides a visual overview of all treatment comparisons included in the network meta-analysis. Node size is proportional to the total sample size of patients receiving each intervention, while the thickness of connecting lines reflects the number of studies contributing direct comparisons. Model convergence for all outcome measures was assessed using trace plots and posterior density plots (Supplementary material 3). The results demonstrated substantial overlap among chains and stable iteration processes, although posterior density distributions for certain outcomes—such as serum 25-(OH)D3, BALP, and serum phosphorus—exhibited right-skewness, indicating the presence of moderate heterogeneity within the model. The BGR diagnostic plots (Supplementary material 4) further showed that the potential scale reduction factor (PSRF) for all parameters was equal to 1, with both the median and 97.5th percentile of the shrink factors approaching unity, confirming satisfactory model convergence. Based on the posterior distributions, SUCRA values were calculated for each intervention across the five outcome measures, and treatments were subsequently ranked according to the area under the cumulative ranking probability curves. Detailed ranking results are presented in Supplementary material 5.

#### 3.5.1 25-(OH)D_3_

A total of seven studies reported serum 25-(OH)D_3_ levels, encompassing 593 participants. The evaluated interventions included vitamin D_3_ combined with calcium/zinc gluconate oral solution (Vit-D_3_ + Ca/Zn-Glu; one study), Shiwei Pugai granules (Vit-D_3_ + SWPG-G; one study), vitamin D-phosphate calcium gluconate tablets (Vit-D_3_ + D_2_-CaP/Gluc; one study), Xinjia Yupingfeng decoction (Vit-D_3_ + XJYPF-T; one study), Saccharomyces boulardii (Vit-D_3_ + S. boulardii; one study), alfacalcidol (Vit-D_3_ + Alfa; one study), and calcium carbonate–vitamin D_3_ granules (Vit-D_3_ + CaCO_3_-D_3_-G; one study).

SUCRA analysis indicated that vitamin D3 combined with Xinjia Yupingfeng decoction (Vit-D3 + XJYPF-T) had the highest probability of being the most effective intervention for improving serum 25-(OH)D3 levels (SUCRA = 96.4%), whereas vitamin D3 monotherapy ranked lowest (SUCRA = 0.0%).

Based on SUCRA rankings, the interventions were ordered from highest to lowest efficacy as follows: Vit-D3 + XJYPF-T (96.4%) > Vit-D3 + S-boulardii (79.0%) > Vit-D3 + CaCO3-D3-G (77.1%) > Vit-D3 + SWPG-G (56.9%) > Vit-D3 + D_2_-CaP/Gluc (47.6%) > Vit-D3 + Ca/Zn-Glu (28.6%) > Vit-D3 + Alfa (14.3%) > Vit-D3 alone (0.0%).

#### 3.5.2 BALP

Seven studies reported BALP levels, comprising a total of 658 participants. The evaluated interventions included vitamin D3 combined with calcium/zinc gluconate oral solution (Vit-D3 + Ca/Zn-Glu; one study), Shiwei Pugai granules (Vit-D3 + SWPG-G; one study), Sijunzi decoction (Vit-D3 + SJZ-T; one study), vitamin D-phosphate calcium gluconate tablets (Vit-D3 + D_2_-CaP/Gluc; one study), calcitriol (Vit-D3 + CAL; two studies), Saccharomyces boulardii (Vit-D3 + S-boulardii; one study), and alfacalcidol (Vit-D3 + Alfa; one study).

SUCRA analysis indicated that vitamin D3 combined with Saccharomyces boulardii (Vit-D3 + S-boulardii) had the highest probability of being the most effective intervention in reducing BALP levels (SUCRA = 96.4%), whereas vitamin D3 combined with Sijunzi decoction (Vit-D3 + SJZ-T) ranked lowest (SUCRA = 0.0%).

Based on SUCRA rankings, the interventions were ordered from highest to lowest efficacy in BALP improvement as follows: Vit-D3 + S-boulardii (98.2%) > Vit-D3 + CAL (82.6%) > Vit-D3 + D_2_-CaP/Gluc (69.2%) > Vit-D3 + Alfa (47.1%) > Vit-D3 + Ca/Zn-Glu (36.1%) > Vit-D3 alone (16.8%) > Vit-D3 + SJZ-T (0.0%).

#### 3.5.3 Serum calcium

Six studies reported serum calcium levels, encompassing a total of 434 participants. The evaluated interventions included vitamin D3 combined with Shiwei Pugai granules (Vit-D3 + SWPG-G; one study), vitamin D-phosphate calcium gluconate tablets (Vit-D3 + D2-CaP/Gluc; one study), calcitriol (Vit-D3 + CAL; two studies), Saccharomyces boulardii (Vit-D3 + S-boulardii; one study), and calcium carbonate-vitamin D3 granules (Vit-D3 + CaCO3-D3-G; one study).

SUCRA analysis suggested that vitamin D3 combined with Shiwei Pugai granules (Vit-D3 + SWPG-G) had the highest probability of being the most effective intervention for improving serum calcium levels (SUCRA = 94.5%), whereas vitamin D3 monotherapy ranked lowest (SUCRA = 0.1%).

Based on SUCRA rankings, the interventions were ordered from highest to lowest efficacy in improving serum calcium as follows: Vit-D3 + SWPG-G (94.5%) > Vit-D3 + CAL (73.9%) > Vit-D3 + D_2_-CaP/Gluc (55.8%), Vit-D3 + S-boulardii (38.5%) > Vit-D3 + CaCO3-D3-G (37.2%) > Vit-D3 alone (0.1%).

#### 3.5.4 Serum phosphorus

Six studies reported serum phosphorus levels, encompassing a total of 434 participants. The evaluated interventions included vitamin D3 combined with Shiwei Pugai granules (Vit-D3 + SWPG-G; one study), vitamin D-phosphate calcium gluconate tablets (Vit-D3 + D_2_-CaP/Gluc; one study), calcitriol (Vit-D3 + CAL; two studies), Saccharomyces boulardii (Vit-D3 + S-boulardii; one study), and calcium carbonate-vitamin D3 granules (Vit-D3 + CaCO_3_-D_3_-G; one study).

SUCRA analysis suggested that vitamin D_3_ combined with Shiwei Pugai granules (Vit-D3 + SWPG-G) had the highest probability of being the most effective intervention for improving serum phosphorus levels (SUCRA = 85.9%), whereas vitamin D3 monotherapy ranked lowest (SUCRA = 0.0%).

Based on SUCRA rankings, the interventions were ordered from highest to lowest efficacy in improving serum phosphorus as follows: Vit-D_3_ + SWPG-G (85.9%) > Vit-D3 + D_2_-CaP/Gluc (82.7%) > Vit-D3 + S. boulardii (67.2%) > Vit-D3 + CAL (41.2%) > Vit-D3 + CaCO3-D3-G (23.0%) > Vit-D3 alone (0.0%).

#### 3.5.5 Adverse reactions

Six studies reported the occurrence of adverse reactions, encompassing a total of 568 participants (Table 2). The evaluated interventions included vitamin D3 combined with calcium–zinc gluconate oral solution (Vit-D3 + Ca/Zn-Glu; one study), Sijunzi decoction (Vit-D3 + SJZ-T; one study), Shiwéi Pugai granules (Vit-D3 + SWPG-G; one study), Saccharomyces boulardii (Vit-D3 + S. boulardii; one study), alfacalcidol (Vit-D3 + Alfa; one study), and calcium carbonate–vitamin D3 granules (Vit-D3 + CaCO_3_-D_3_-G; one study).

**Table 2:** Characteristics of included studies.

References	Intervention (C/I)	Safety reporting status	Description (C/I)
Jiang (19)	C: Vitamin D_3_ I: C + Ca/Zn-Glu	Adverse events reported	C: Nausea and vomiting (2 cases), constipation (0 case), skin itching (1 case), chapped lips (1 case), loss of appetite (1 case). (5/49 = 10.20%) I: Nausea and vomiting (1 case), constipation (1 case), skin itching (2 cases), chapped lips (3 cases), loss of appetite (0 case). (7/49 = 14.29%)
Zhang and Gao (17)	C: Vitamin D_3_ I: C + SJZ-T	Adverse events reported	C: Vomiting (2 cases), diarrhea (1 case), constipation (1 case), (4/56 = 7.14%) I: Vomiting (2 cases), diarrhea (2 cases), constipation (2 cases), (6/50 = 12%)
Xu (16)	C: Vitamin D_3_ I: C + SWPG-G	Adverse events reported	C: Nausea and vomiting (2 cases), belching (1 case), constipation (2 cases), hypercalcemia (1 case), (6/41 = 14.63%) I: Nausea and vomiting (1 case), belching (1 case), constipation (1 case), hypercalcemia (0 case), (3/41 = 7.32%)
Liu (15)	C: Vitamin D_3_ I: C + S-boulardii	Adverse events reported	C: Skin itching (1 case), diarrhea (0 case), nausea and vomiting (0 case), taste (1 case), (2/30 = 13.33%) I: Skin itching (1 case), diarrhea (1 case), nausea and vomiting (1 case), dry mouth (1 case), (4/30 = 6.67%)
Hu (21)	C: Vitamin D_3_ I: C + Alfa	Adverse events reported	C: Irritability (4 cases), excessive sweating (2 cases), night terrors (5 cases), pillow baldness (2 cases), (13/81 = 16.04%) I: Irritability (2 cases), excessive sweating (1 case), night terrors (1 case), pillow baldness (1 case), (5/81 = 6.17%)
Meng and Jiao (18)	C: Vitamin D_3_ I: C + CaCO-D3-G	Adverse events reported	C: Constipation (2 cases), belching (1 case), dizziness (1 case), (4/31 = 12.90%) I: Constipation (1 case), belching (1 case), dizziness (1 case), (3/29 = 10.34%)

SUCRA analysis indicated that vitamin D3 combined with S. boulardii had the highest probability of being the safest regimen, as reflected by the lowest SUCRA value for adverse reactions (SUCRA = 21.7%), whereas vitamin D3 combined with alfacalcidol ranked lowest in terms of safety (SUCRA = 88.0%).

Based on SUCRA values, the interventions were ranked in descending order of adverse reaction risk as follows: Vit-D3 + Alfa (88.0%) > Vit-D3 + SWPG-G (76.9%) > Vit-D3 + CaCO3-D3-G (57.8%) > Vit-D3 alone (48.3%) > Vit-D3 + Ca/Zn-Glu (32.3%) > Vit-D3 + S-boulardii (21.7%).

### 3.6 Sensitivity analysis

To assess the robustness of the primary findings of this network meta-analysis, leave-one-out sensitivity analyses were conducted for five key outcomes: serum 25-(OH)D3, BALP, serum phosphorus, serum calcium, and adverse events (Supplementary material 6).

1. 25-(OH)D3: The pooled MD for serum 25-(OH)D3 was 15.1 (95% CI: 10.0–20.3). Sequential exclusion of individual studies resulted in modest fluctuations in the pooled estimate, ranging from −1.0 to +2.2. The largest increase was observed after removal of Hu (21) (Δ = +2.2), whereas exclusion of Wang et al. (14) produced the greatest attenuation (Δ = −1.1). Notably, all recalculated 95% confidence intervals remained above the null value, and statistical significance was preserved, indicating a high degree of robustness.
2. BALP: The overall pooled MD for BALP was −28.4 (95% CI: −40.0 to −16.8). Leave-one-out analyses yielded effect size changes between −3.9 and +3.9. Exclusion of Jiang et al. (19) resulted in the largest shift (MD = −32.3; Δ = −3.9), suggesting a tendency toward a more pronounced negative effect, whereas omission of Liu (15) led to a relative attenuation (MD = −25.6; Δ = +2.8). Nevertheless, none of the recalculated confidence intervals crossed the null, and the majority of exclusions produced only minor variations, supporting the stability of the BALP findings.
3. Serum phosphorus: The pooled MD for serum phosphorus was 0.22 (95% CI: 0.16–0.27). Sequential exclusion of individual studies resulted in minimal changes, ranging from −0.02 to +0.02. All recalculated estimates remained statistically significant, with narrow confidence intervals that did not cross the null, indicating that the overall result was stable and not driven by any single study.
4. Serum calcium: The pooled MD for serum calcium was 0.22 (95% CI: 0.18–0.25). Leave-one-out analyses demonstrated very limited variability (−0.01 to +0.02). Across all iterations, the direction and statistical significance of the effect remained unchanged, and none of the confidence intervals crossed the null, confirming the robustness of the serum calcium outcome.
5. Adverse events: The pooled RR for adverse events was 0.85 (95% CI: 0.53–1.36), indicating no statistically significant difference between intervention and control groups, as the confidence interval encompassed unity. Sequential exclusion of individual studies produced RR changes ranging from −0.29 to +0.11, with the largest increase observed after removal of Hu (21) (Δ = +0.29). Importantly, all recalculated confidence intervals consistently crossed the null value (RR = 1.0), and the overall interpretation remained unchanged, suggesting stable results for adverse events.

Overall, leave-one-out sensitivity analyses across all five outcomes did not identify any single study exerting a decisive influence on the pooled estimates. These findings collectively support the robustness and reliability of the main conclusions of this network meta-analysis.

## 4 Discussion

In this study, we performed a network meta-analysis to systematically assess the efficacy and safety of nine vitamin D–based combination therapies compared with vitamin D3 monotherapy in children with rickets, incorporating evidence from ten randomized controlled trials. The primary outcomes included serum 25-(OH)D3, BALP, serum calcium, serum phosphorus, and adverse events, collectively capturing key dimensions of bone metabolic status and treatment safety. The pooled analyses demonstrated that, relative to vitamin D3 alone, combination therapies were associated with statistically significant improvements across major biochemical markers of bone metabolism, without a corresponding increase in the risk of adverse events. Furthermore, by integrating both direct and indirect evidence, the network meta-analysis revealed heterogeneity in the relative efficacy of individual combination regimens across outcome domains, suggesting that distinct therapeutic strategies may confer advantages in different treatment dimensions. These findings extend current understanding of the therapeutic spectrum of vitamin D–based combination interventions and provide preliminary evidence to support stratified and individualized treatment approaches guided by specific clinical objectives. The following sections elaborate on these principal observations, situating them within the context of existing evidence while addressing potential mechanisms, clinical implications, sources of heterogeneity, and study limitations.

The pairwise meta-analyses consistently demonstrated that, compared with vitamin D3 monotherapy, vitamin D-based combination therapies were associated with statistically significant improvements across multiple key markers of bone metabolism in children with rickets. Specifically, combination regimens resulted in higher serum 25-(OH)D3 concentrations, lower levels of BALP, and improved serum calcium and phosphorus levels (all *P* < 0.05). Importantly, pooled analyses did not indicate a significant increase in the risk of adverse events associated with combination therapy (*P* > 0.05). Taken together, these findings suggest that, within the current evidence framework, combination interventions confer more comprehensive benefits in improving bone metabolic status, while maintaining a short-term safety profile comparable to that of vitamin D3 monotherapy. From an evidence-based perspective, these findings support the theoretical rationale for implementing coordinated, multi-component interventions in rickets, a disorder characterized by multifactorial etiologies and involvement of multiple physiological pathways. Vitamin D3 monotherapy primarily targets the correction of vitamin D deficiency, which represents a central pathological basis of the disease; however, its clinical effectiveness may be influenced by several factors, including intestinal absorption efficiency, metabolic activation capacity, and the availability of calcium and phosphorus. By incorporating interventions with distinct mechanisms of action, combination therapies may theoretically exert effects across multiple critical pathways simultaneously, thereby facilitating improvements in bone metabolic status at a systemic level. For example, the addition of calcium or phosphorus supplements may directly replenish substrates required for bone mineralization (23, 24); active vitamin D analogs may partially mitigate limitations related to impaired metabolic activation (25); probiotics may indirectly enhance nutrient absorption by improving gastrointestinal function (26); and traditional herbal formulations may contribute to a more favorable internal milieu through holistic regulation of physiological balance, potentially facilitating nutrient utilization (27). The present results indicate that such multi-pathway combination interventions exert synergistic effects on short-term improvements in biochemical markers of bone metabolism, rather than merely additive or mutually offsetting effects. With respect to safety, combination therapies were not associated with a higher risk of adverse events compared with vitamin D3 monotherapy, a finding of notable clinical relevance. Given that rickets typically requires sustained intervention and follow-up, the safety profile of a therapeutic regimen is closely linked to its clinical acceptability and patient adherence. Our findings suggest that, within the dosage ranges and treatment durations investigated, the introduction of combination therapies did not impose an increased short-term safety burden. Nevertheless, this conclusion warrants cautious interpretation. On the one hand, heterogeneity exists across studies in terms of adverse event monitoring and reporting standards, and most reported events were mild and nonspecific, predominantly involving gastrointestinal symptoms. On the other hand, current evidence is limited by the lack of systematic evaluation of potential long-term risks, particularly those associated with prolonged use of active vitamin D analogs and calcium supplementation. Accordingly, existing evidence primarily supports the relative short-term tolerability of combination therapies, whereas their long-term safety remains to be established through studies with longer follow-up durations and more rigorous designs.

In summary, moving beyond the correction of isolated vitamin D deficiency toward a comprehensive management strategy based on multi-pathway synergy, this study provides supportive biochemical evidence for combination interventions. These findings suggest that, in clinical practice, vitamin D–based combination therapies may be considered for children who respond suboptimally to vitamin D3 monotherapy or present with conditions such as prematurity, malabsorption, or severe vitamin D deficiency. Nevertheless, their clinical application should be individualized and guided by patient-specific factors, study quality, and the availability of long-term outcome data.

One of the most informative findings of the present study lies in the systematic ranking of the relative efficacy of different vitamin D-based combination therapies using a network meta-analytic framework. The SUCRA estimates indicated that no single combination regimen demonstrated uniform superiority across all evaluated outcomes; instead, distinct interventions exhibited differentiated strengths across specific efficacy domains. Importantly, these rankings should not be interpreted as a simplistic hierarchy of overall effectiveness. Rather, they offer an evidence-based perspective on the heterogeneous roles that different therapeutic pathways may play in the management of rickets, thereby providing meaningful insights into their distinct effect profiles and informing individualized clinical decision-making. With respect to improving serum 25-(OH)D3 concentrations, the combination of vitamin D3 with Xinjia Yupingfeng Decoction demonstrated a relatively high probability of ranking favorably. Xinjia Yupingfeng Decoction, a modified herbal formulation employed in several of the clinical trials included in this study, consists of *Astragalus membranaceus, Atractylodes macrocephala, Saposhnikovia divaricata*, Os Draconis (fossilized bone), Concha Ostreae (oyster shell), *Angelica sinensis*, and *Citrus reticulata pericarpium*, among other components. Contemporary pharmacological investigations suggest that Os Draconis and Concha Ostreae are rich in organically derived calcium of biological origin, characterized by favorable bioavailability and potential benefits for enhancing bone toughness and structural integrity (28). These properties imply that the formulation may exert dual mechanistic effects: indirectly facilitating vitamin D utilization through modulation of systemic physiological balance, and directly contributing bioavailable calcium substrates essential for bone mineralization. Collectively, these features provide a biologically plausible rationale for its adjunctive use in the management of disorders characterized by impaired bone metabolism. Serum 25-(OH)D3 serves as a key indicator of vitamin D nutritional status and systemic availability (29). The observed relative advantage of this regimen suggests that the herbal formulation may not act by directly supplying vitamin D, but rather by modulating overall physiological conditions that facilitate vitamin D absorption, transport, and utilization (30). Another formulation evaluated in this study, Sijunzi Decoction, is a classical prescription traditionally indicated for the reinforcement of digestive and metabolic function. It comprises *Panax ginseng, Atractylodes macrocephala, Poria cocos*, and *Glycyrrhiza uralensis*. In the management of vitamin D deficiency rickets, Sijunzi Decoction may augment therapeutic outcomes by enhancing gastrointestinal functional capacity, thereby promoting the absorption and bioavailability of calcium, phosphorus, and vitamin D (31). From a translational perspective, this suggests that the physiological status of the digestive–absorptive system—conceptually aligned with the traditional notion of “spleen function”—may constitute a critical mechanistic interface linking herbal interventions to the modulation of bone metabolism. This pattern is consistent with a potential indirect pathway through which nutritional status may be enhanced. This pattern is consistent with a potential indirect pathway through which nutritional status may be enhanced. With respect to the BALP, a marker reflecting bone turnover status, the combination of vitamin D3 with Saccharomyces boulardii demonstrated a relatively pronounced advantage. BALP is widely recognized as an indicator of osteoblastic activity and overall bone metabolic dynamics, and a reduction in its level generally suggests attenuation of abnormally accelerated bone turnover (32, 33). This observation is clinically informative, as it implies a potential association between improvements in intestinal function and the regulation of bone metabolism. In recent years, increasing attention has been directed toward the interplay between the gut microbiota and bone metabolism. Probiotics have been proposed to influence skeletal homeostasis indirectly by enhancing intestinal barrier integrity, alleviating low-grade inflammation, facilitating mineral absorption, and modulating immune-related pathways (34–37). Although these mechanisms remain to be fully elucidated, the relative advantage of this combination therapy with respect to BALP observed in the present study provides suggestive evidence supporting the role of gut function modulation as an adjunctive strategy in the management of rickets. With regard to the improvement of serum calcium and phosphate levels, the combination of vitamin D3 with Siwei Pugai Granules exhibited a comparatively high potential benefit. This finding is consistent with established clinical and pharmacological principles, whereby, under the regulatory influence of vitamin D, direct supplementation with calcium and phosphate facilitates more rapid correction of hypocalcaemia or hypophosphatemia, thereby providing essential substrates for bone mineralization (38). In addition, although combination regimens involving active vitamin D analogs did not rank highest for any single outcome in the present analysis, their clinical relevance in specific patient populations should not be overlooked. In children with immature hepatic or renal function, or with impaired metabolic conversion capacity, native vitamin D may not be efficiently transformed into its biologically active forms, whereas active vitamin D analogs can exert regulatory effects directly (39–41). The observed therapeutic effects of such regimens in this study provide evidence-based support for their rational use in well-defined clinical indications.

Overall, the heterogeneous performance of different combination strategies across outcomes suggests that rickets management should not rely on a uniform therapeutic approach, but rather be tailored according to individual clinical characteristics and prevailing treatment priorities. Aligning therapeutic choices with specific efficacy targets may enhance treatment efficiency while minimizing unnecessary pharmacological exposure. Such a treatment goal–oriented combination strategy appears more consistent with the complex pathophysiology of rickets than non-stratified treatment paradigms and offers a clear framework for the future optimization of therapeutic regimens.

The extremely high heterogeneity observed in this study (with I^2^ values reaching up to 98.7%) represents a central issue that warrants careful consideration when interpreting the findings. Comprehensive evaluation suggests that this heterogeneity primarily arises from substantial differences across included studies at three levels: intervention characteristics, population features, and methodological design. First, the pronounced heterogeneity of the interventions themselves constitutes the most fundamental source of the observed variability. The combination regimens included in this study encompassed markedly distinct therapeutic pathways, ranging from direct supplementation of minerals such as calcium and phosphorus, to the use of active vitamin D analogs that bypass metabolic conversion barriers, as well as strategies that indirectly influence bone metabolism by modulating the intestinal milieu or overall physiological status. These interventions do not target the same pathophysiological processes and therefore differ fundamentally in their onset of action, primary target outcomes, and duration of effect. For example, direct calcium or phosphorus supplementation can rapidly correct serum electrolyte abnormalities but does not directly modify vitamin D nutritional reserves (42). In contrast, combination strategies aimed at improving absorptive capacity or global metabolic conditions tend to exert slower effects on bone metabolic markers or vitamin D levels, yet may operate in a more integrative manner (43). Such mechanistic divergence inevitably leads to variability in effect estimates across studies assessing identical outcomes and, in turn, contributes substantially to the magnitude of statistical heterogeneity observed. Second, differences in study population characteristics—particularly gestational age–related physiological variation—act as important effect modifiers of treatment response. Subgroup analyses in the present study indicate that, within the limits of the available evidence, full-term infants exhibit a greater biochemical response to combination therapy than preterm infants. This disparity has a clear physiological basis: preterm infants exhibit immature gastrointestinal development, reduced absorptive surface area, and insufficient bile acid secretion, all of which compromise the absorption of fat-soluble vitamin D (44, 45). In addition, immature hepatic and renal function may limit 25-hydroxylation and 1α-hydroxylation of vitamin D, thereby attenuating its downstream biological effects (7, 46, 47). Concomitant clinical conditions common in preterm infants, such as infection and feeding difficulties, may further disrupt nutrient metabolism and utilization (48, 49). Importantly, however, the preterm subgroup in this analysis was informed by only a single study. Although the sample size was relatively large, it was insufficient to capture the full spectrum of heterogeneity across gestational ages and birth weight categories. Consequently, the current evidence supports the conclusion that preterm infants exhibit a weaker response to combination therapy within the available studies, but does not justify the inference that such interventions are ineffective in this population. This finding underscores the urgent need for high-quality, stratified clinical trials specifically targeting preterm infants. Finally, inter-study differences in treatment duration (ranging from 1 to 6 months), dosing regimens, baseline nutritional status, and laboratory measurement methods may have further amplified overall heterogeneity. These observations highlight the importance of greater standardization in outcome measurement and reporting in future research to enhance comparability and reliability in evidence synthesis.

This study employed a NMA to integrate direct and indirect evidence, enabling the relative ranking of nine combination interventions in a clinical context where head-to-head RCTs comparing multiple regimens are largely unavailable. This approach represents a meaningful methodological advancement in the field. Sensitivity analyses indicated that the principal findings were reasonably robust. Nevertheless, several important limitations should be carefully considered when interpreting the results. (1) Language and international verifiability. Although no language restrictions were imposed during the search process, the studies meeting inclusion criteria were predominantly conducted in Chinese populations and published in Chinese. This distribution reflects the current configuration of the evidence base rather than an *a priori* language constraint. Nevertheless, the predominance of Chinese-language publications may hinder independent verification by non-Chinese-speaking readers and may limit the external generalizability of our findings across diverse healthcare systems and populations. Moreover, given the limited number of included trials, a reliable quantitative assessment of publication bias was not feasible. (2) Limited number and methodological rigor of included studies. Only ten randomized controlled trials were eligible for inclusion, most of which were characterized by modest sample sizes. Critical methodological elements—including randomization procedures, allocation concealment, and blinding—were incompletely reported in several studies, resulting in an overall low certainty of evidence. These limitations reduce the precision of effect estimates and temper the strength of the inferences that can be drawn. (3) Broad age range and unexamined effect modification. The included studies encompassed a wide pediatric age spectrum (3 months to 8 years), spanning infancy, toddlerhood, and early childhood. Vitamin D metabolism, calcium homeostasis, bone turnover dynamics, and therapeutic responsiveness may differ substantially across these developmental stages, rendering age a plausible effect modifier. However, owing to insufficient age-stratified data in the primary trials, neither subgroup analyses nor meta-regression by age could be performed. Accordingly, the present findings should be interpreted as average effects across a heterogeneous pediatric population rather than precise age-specific estimates. (4) Predominant reliance on short-term biochemical outcomes with limited clinically meaningful endpoints. The present analysis primarily evaluated treatment efficacy using biochemical markers, including serum 25-(OH)D3, BALP, calcium, and phosphate levels, while lacking data on skeletal radiographic improvement (e.g., wrist X-ray scoring), anthropometric growth indices (e.g., height- and weight-for-age Z scores), and long-term functional outcomes such as fracture incidence or correction of skeletal deformities. Consequently, whether short-term biochemical normalization translates into sustained and clinically meaningful long-term benefits remains uncertain and warrants further investigation. (5) Inadequate control of potential confounding factors. Both the primary studies and the present secondary analysis were limited in their ability to account for key confounders that may substantially influence treatment response, including baseline severity of vitamin D deficiency, dietary calcium intake, sunlight exposure, and genetic determinants such as polymorphisms in the vitamin D receptor gene.

In light of these limitations, future research should prioritize rigorous study design and transparent reporting, implement age-stratified or individual participant data meta-analytic approaches, expand sample sizes and follow-up durations, incorporate clinically meaningful long-term endpoints, and systematically control for critical confounding variables. Moreover, bilingual dissemination and strengthened international collaboration will be essential to enhance the evidentiary hierarchy and global applicability of the findings.

## 5 Conclusion

This network meta-analysis indicates that vitamin D-based combination therapies are, overall, more effective than vitamin D3 monotherapy in improving biochemical markers of bone metabolism in children with rickets, without evidence of an increased risk of adverse events. Different combination regimens demonstrated distinct advantages across specific outcomes, with traditional herbal formulations and probiotic-based combinations showing particularly favorable performance in selected endpoints. Infant type emerged as an important modifier of treatment response, with term infants deriving more pronounced benefits than preterm infants. Collectively, the available evidence supports the use of individualized, goal-oriented vitamin D combination strategies in clinical practice, especially for term infants with rickets. Nevertheless, these conclusions should be interpreted with caution given the limited number of eligible studies, the generally low methodological quality of the evidence, and the paucity of data in preterm populations. Future research should prioritize well-designed, multicenter randomized controlled trials with larger sample sizes, longer follow-up, and more comprehensive, clinically meaningful outcome measures to substantiate and refine these findings.

## Data Availability

The original contributions presented in the study are included in the article/Supplementary material, further inquiries can be directed to the corresponding author.
