# Supplementary material for: Comprehensive evaluation of the efficacy and safety of different vitamin D combination regimens based on indirect comparisons for children with rickets: a network meta-analysis
Source: Front Nutr. 2026 Apr 8;13:1785775. doi: 10.3389/fnut.2026.1785775 (PMC13099536; doi:10.3389/fnut.2026.1785775)
Supplement: Supplementary file 1 [file Supplementary_file_1.docx]

**Supplement**

Trajectory diagram and density diagram


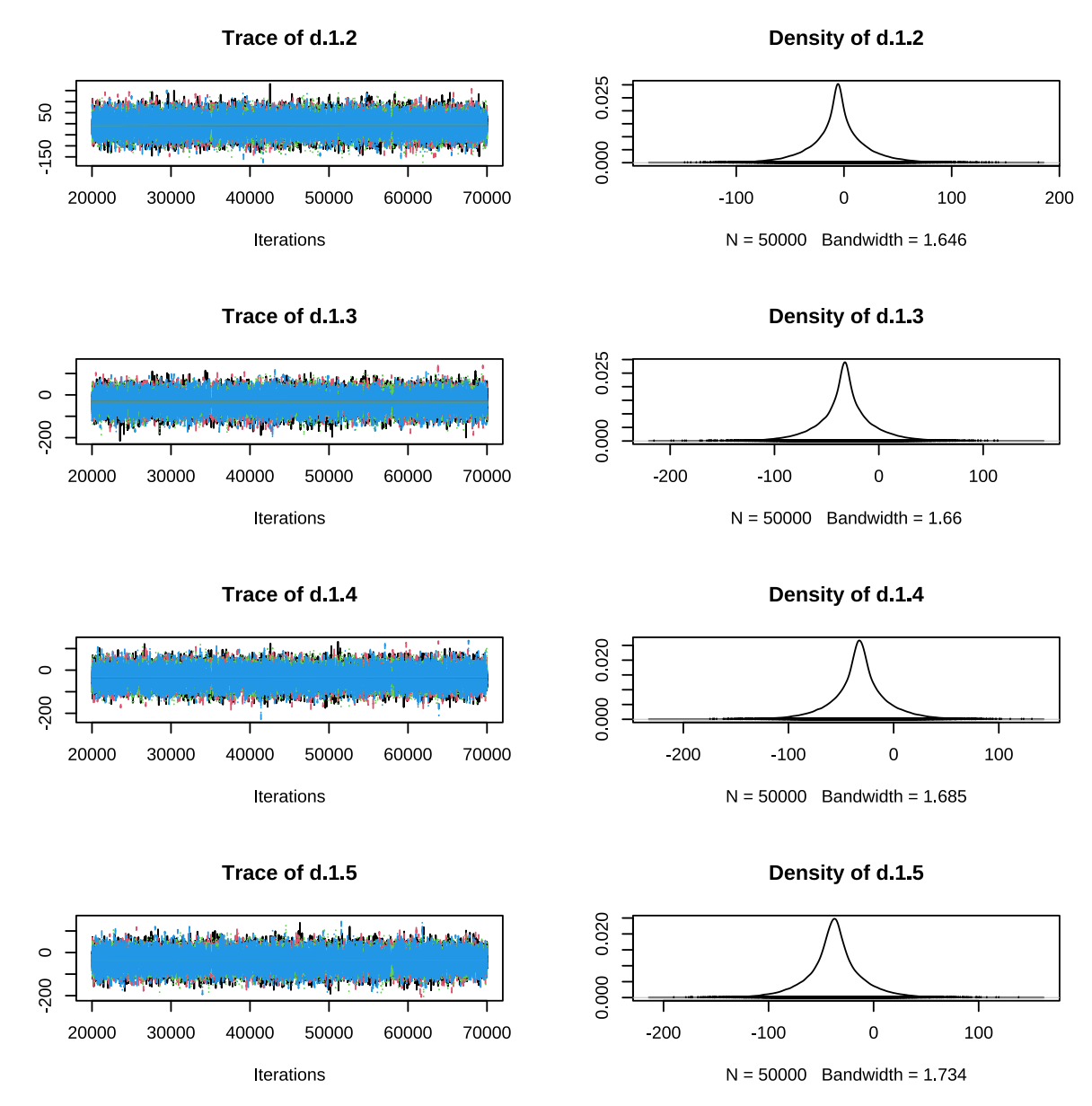

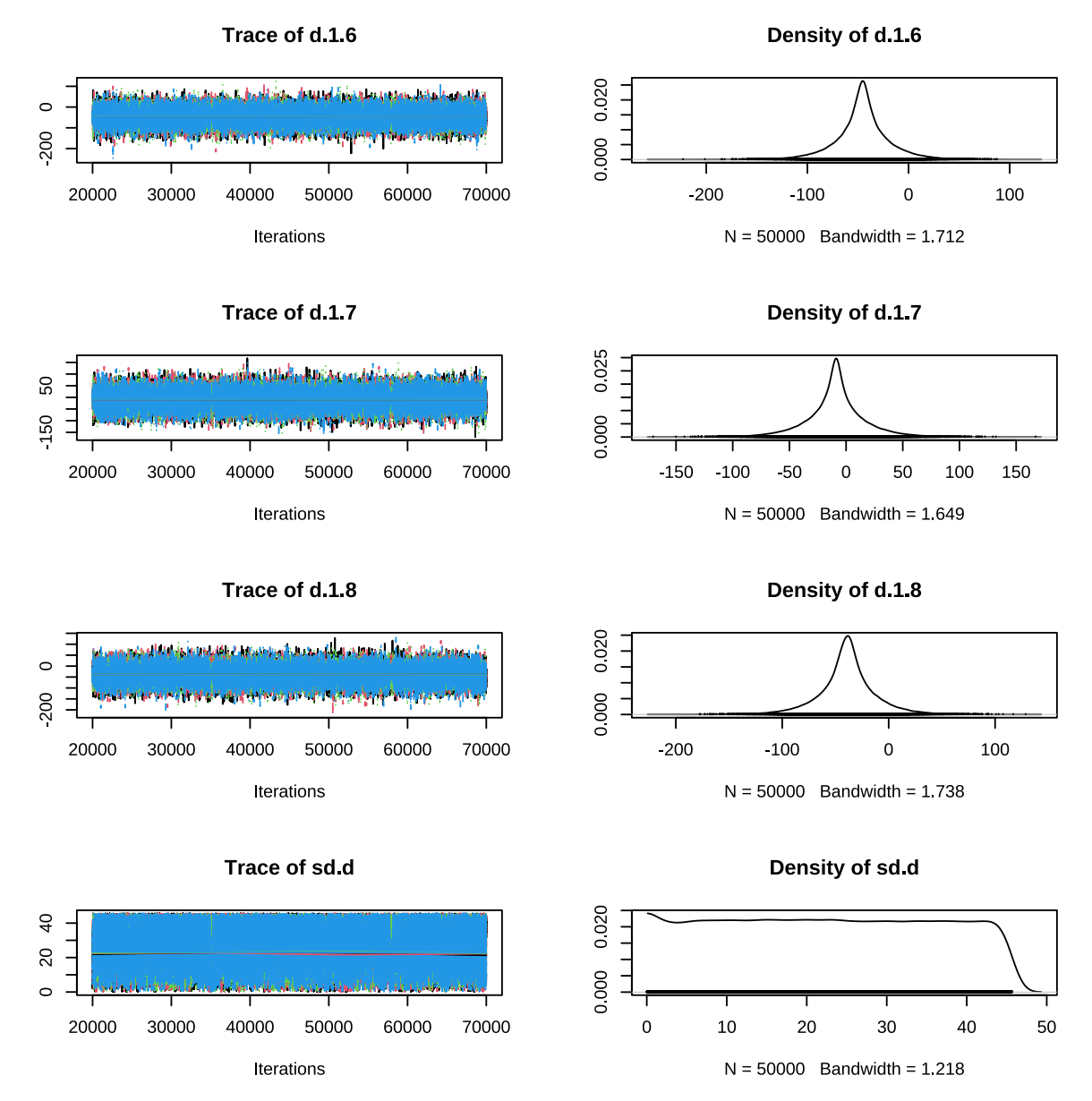


**Figure S1.** **Trajectory diagram and density diagram: 25-(OH)D₃**


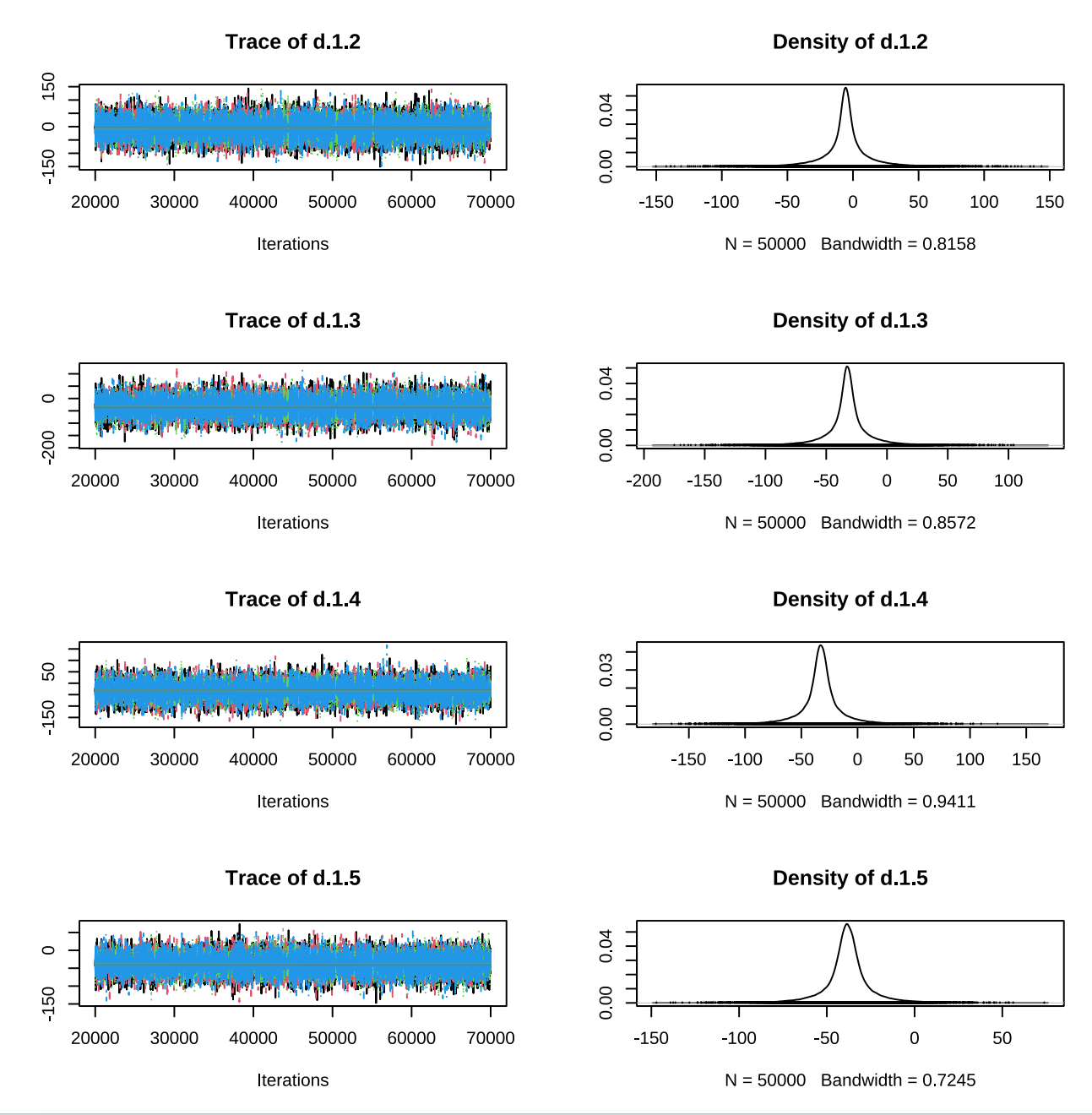


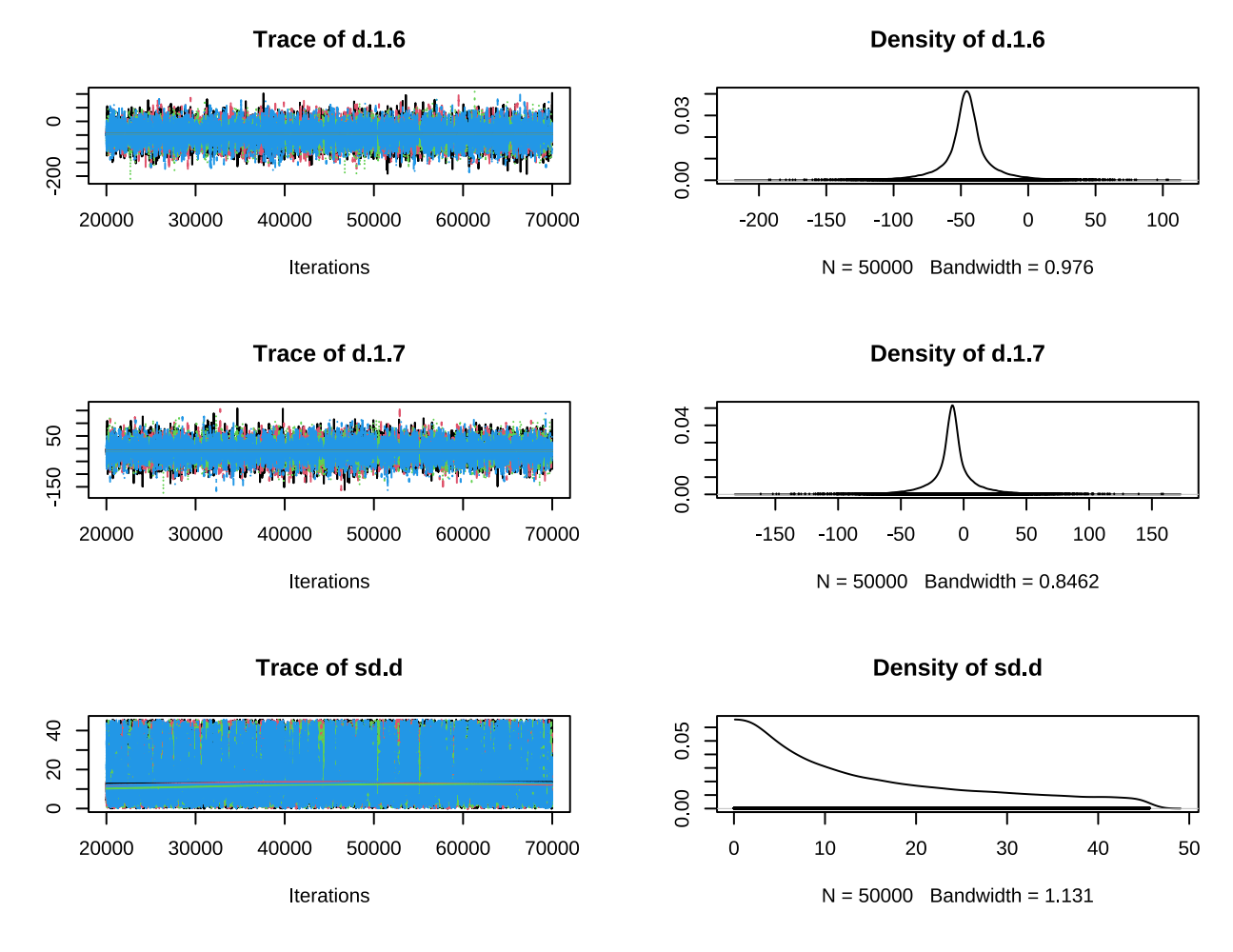


**Figure S2.** **Trajectory diagram and density diagram: BALP**


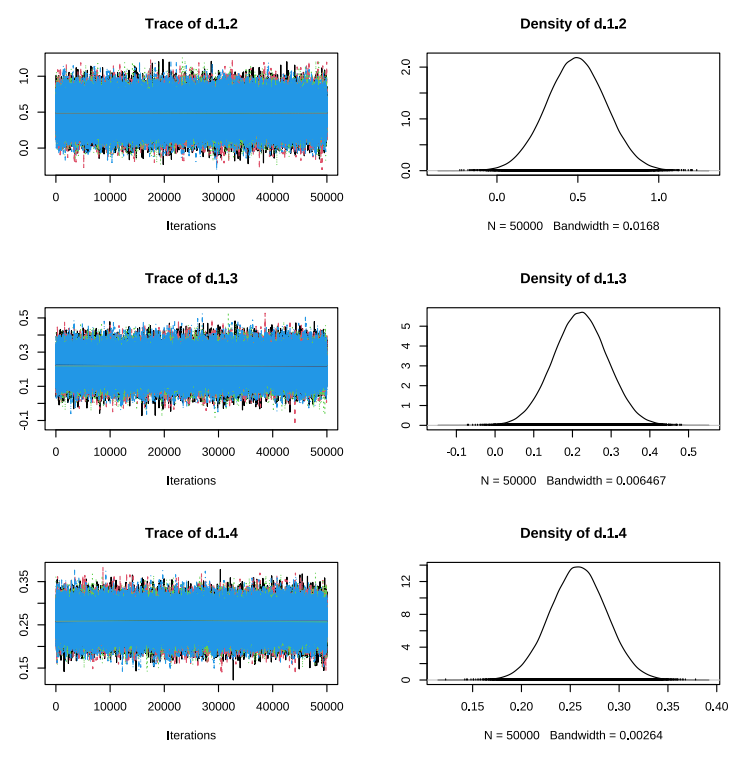


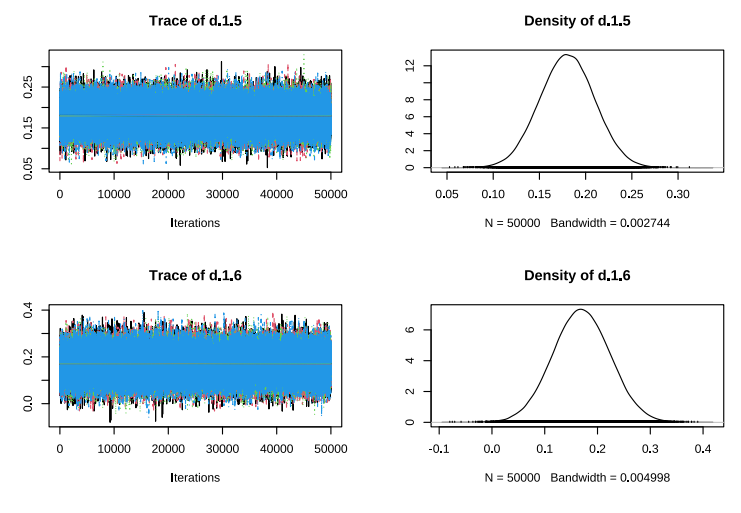


**Figure S3.** **Trajectory diagram and density diagram: Serum calcium**


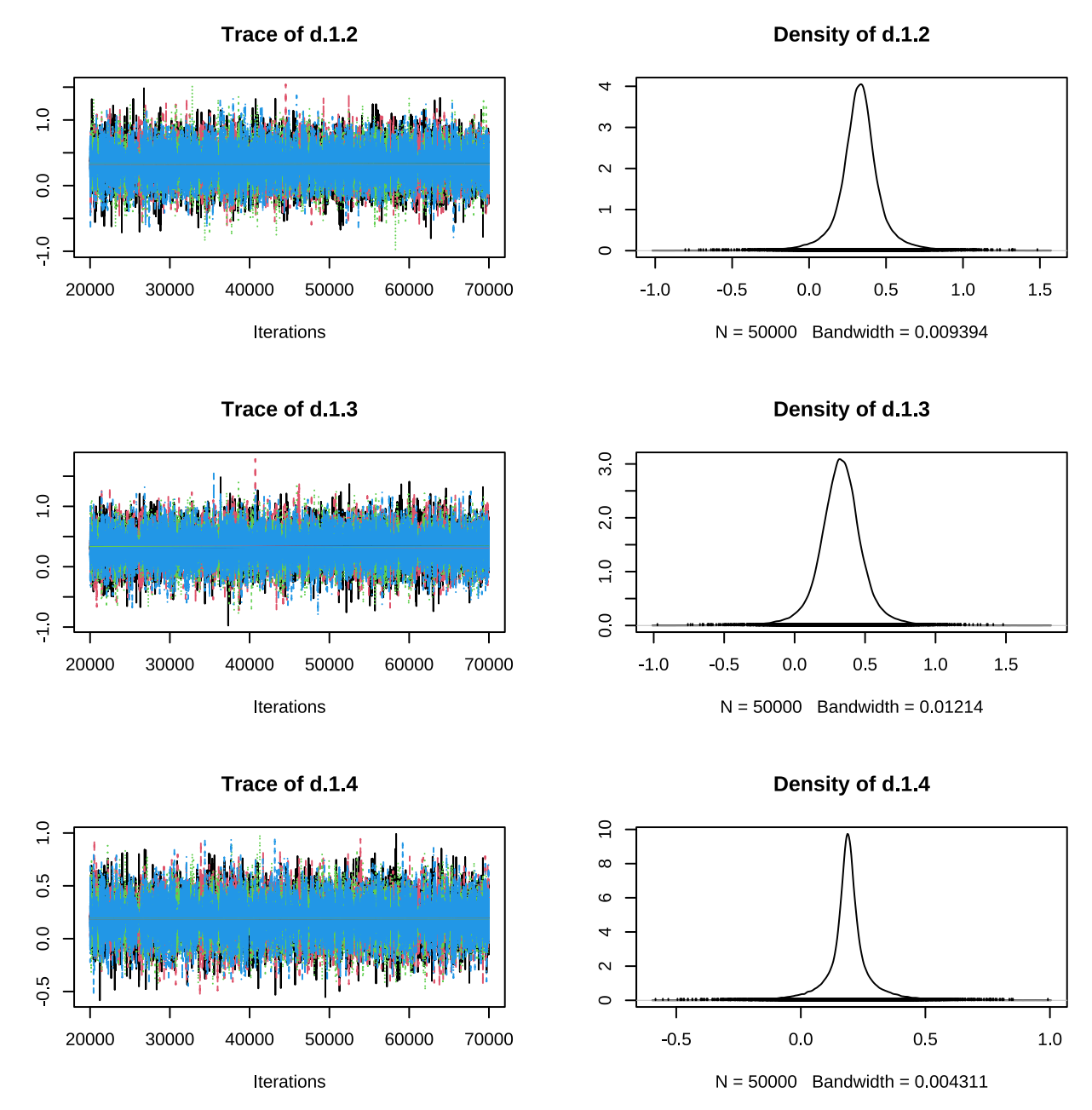


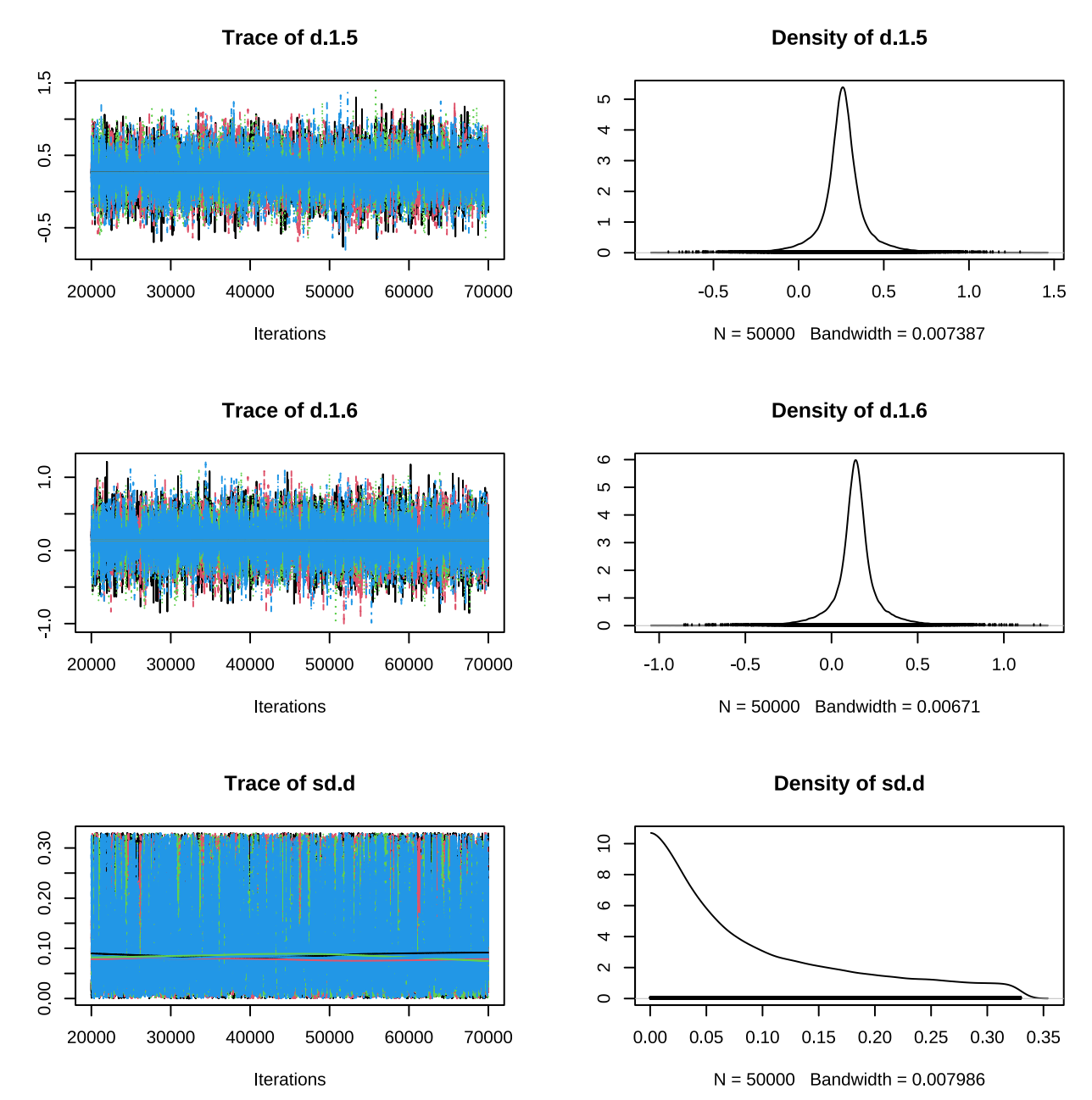


**Figure S4.** **Trajectory diagram and density diagram: Serum phosphorus**


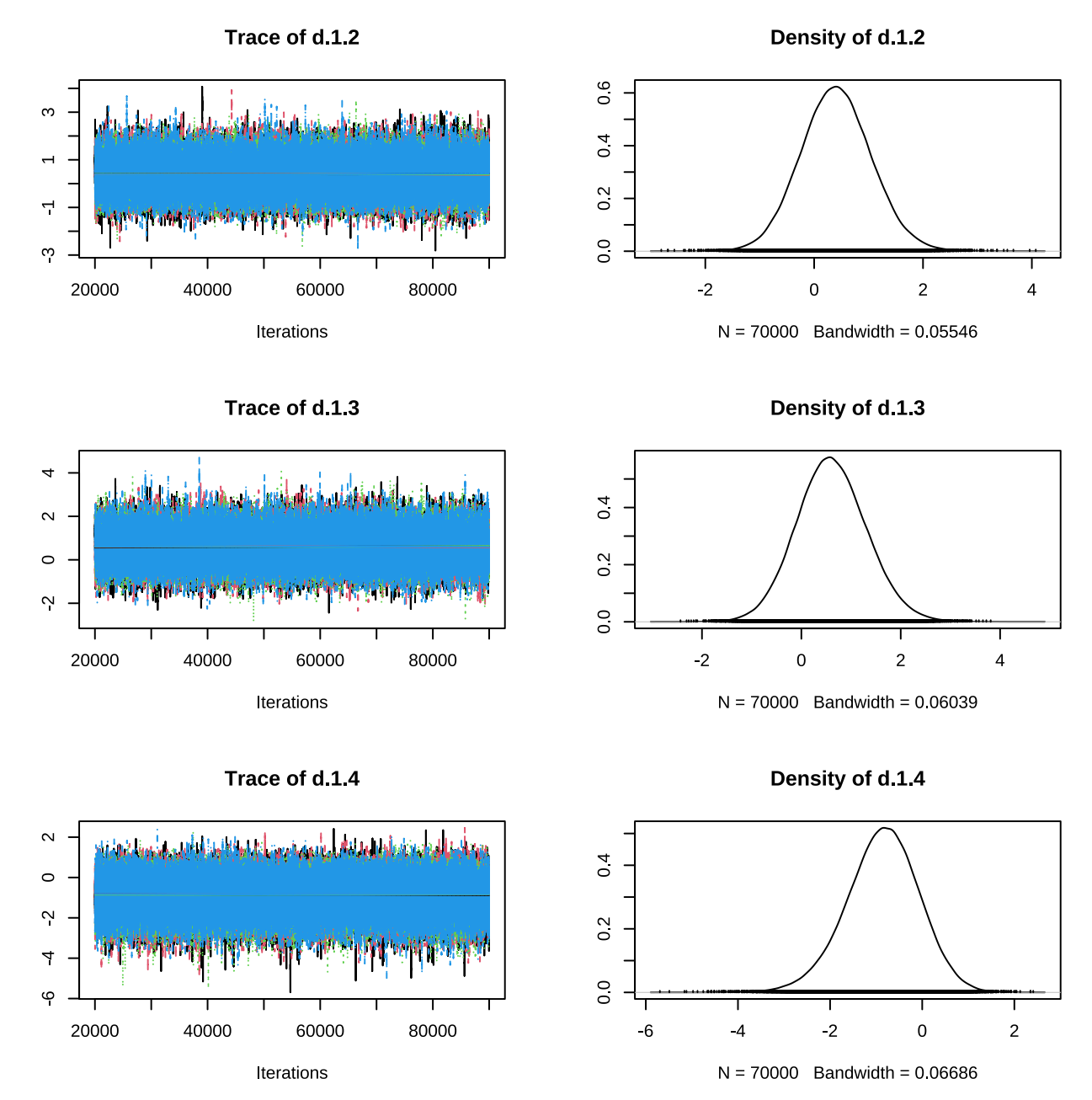

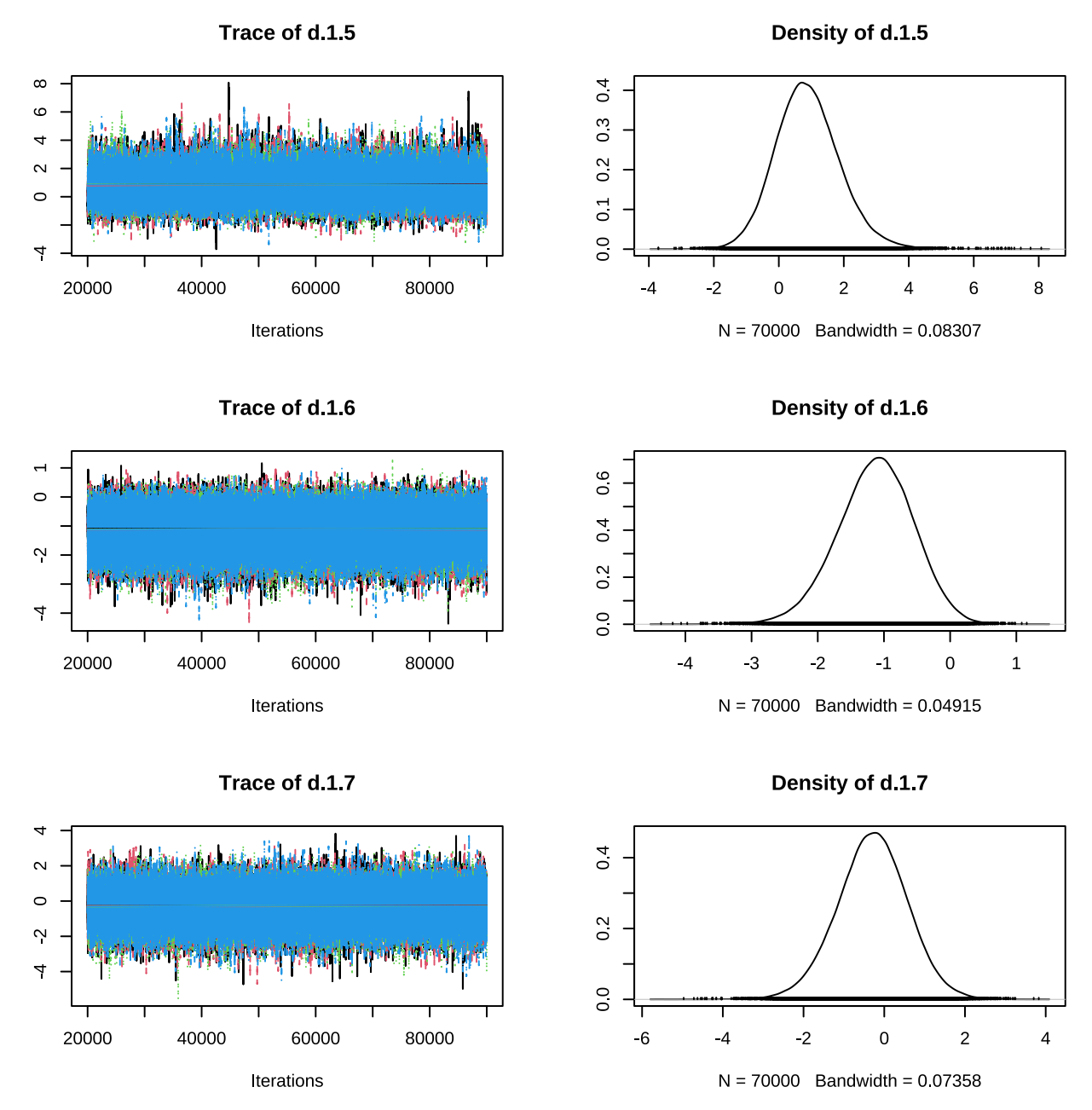


**Figure S5.** **Trajectory diagram and density diagram: Adverse actions**
