# Supplementary material for: Comprehensive evaluation of the efficacy and safety of different vitamin D combination regimens based on indirect comparisons for children with rickets: a network meta-analysis
Source: Front Nutr. 2026 Apr 8;13:1785775. doi: 10.3389/fnut.2026.1785775 (PMC13099536; doi:10.3389/fnut.2026.1785775)
Supplement: Supplementary file 2 [file Supplementary_file_2.docx]

**Supplement**

diagnostic graph


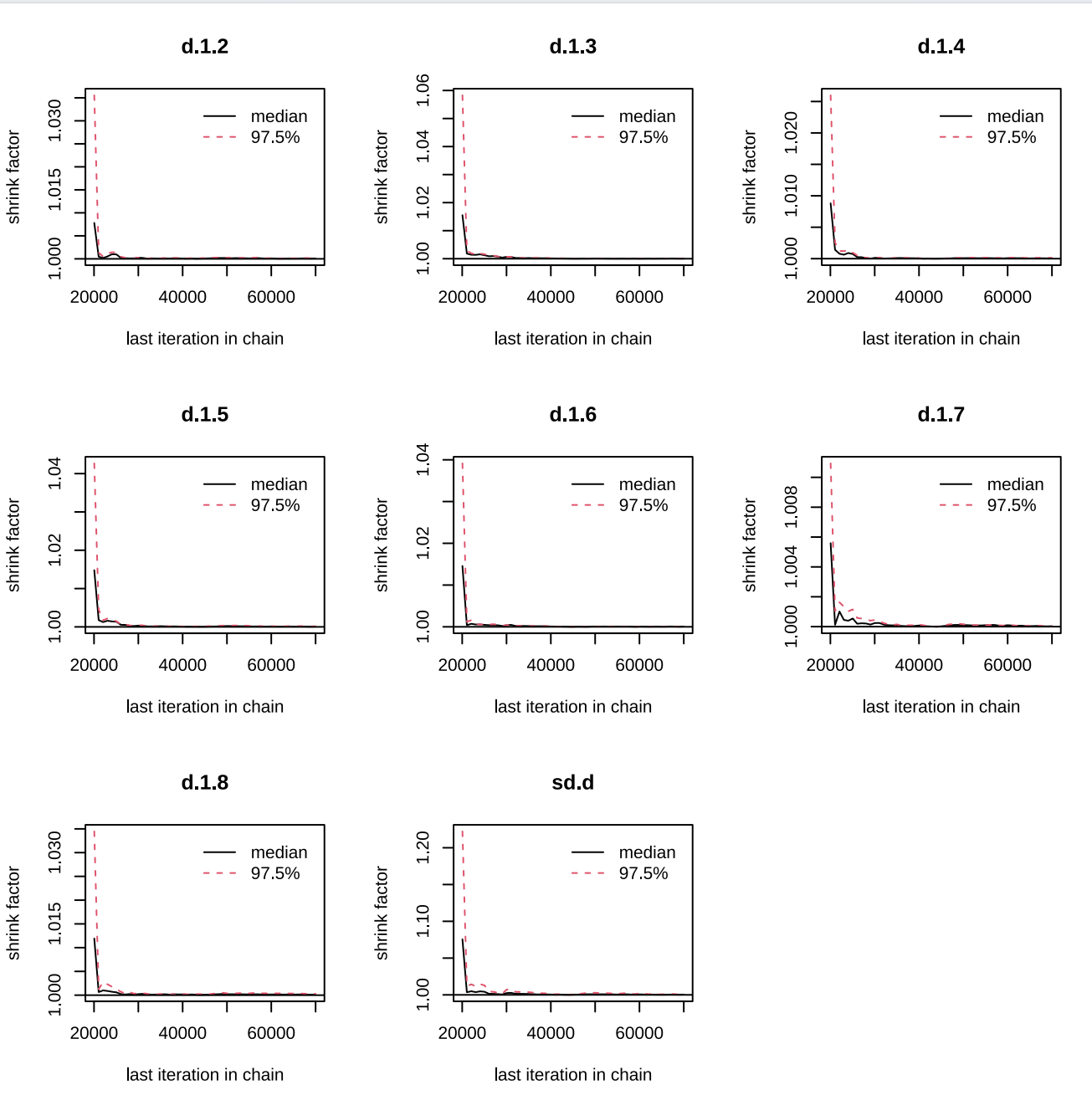


**Figure S1. diagnostic graph:** **25-(OH)D_3_**


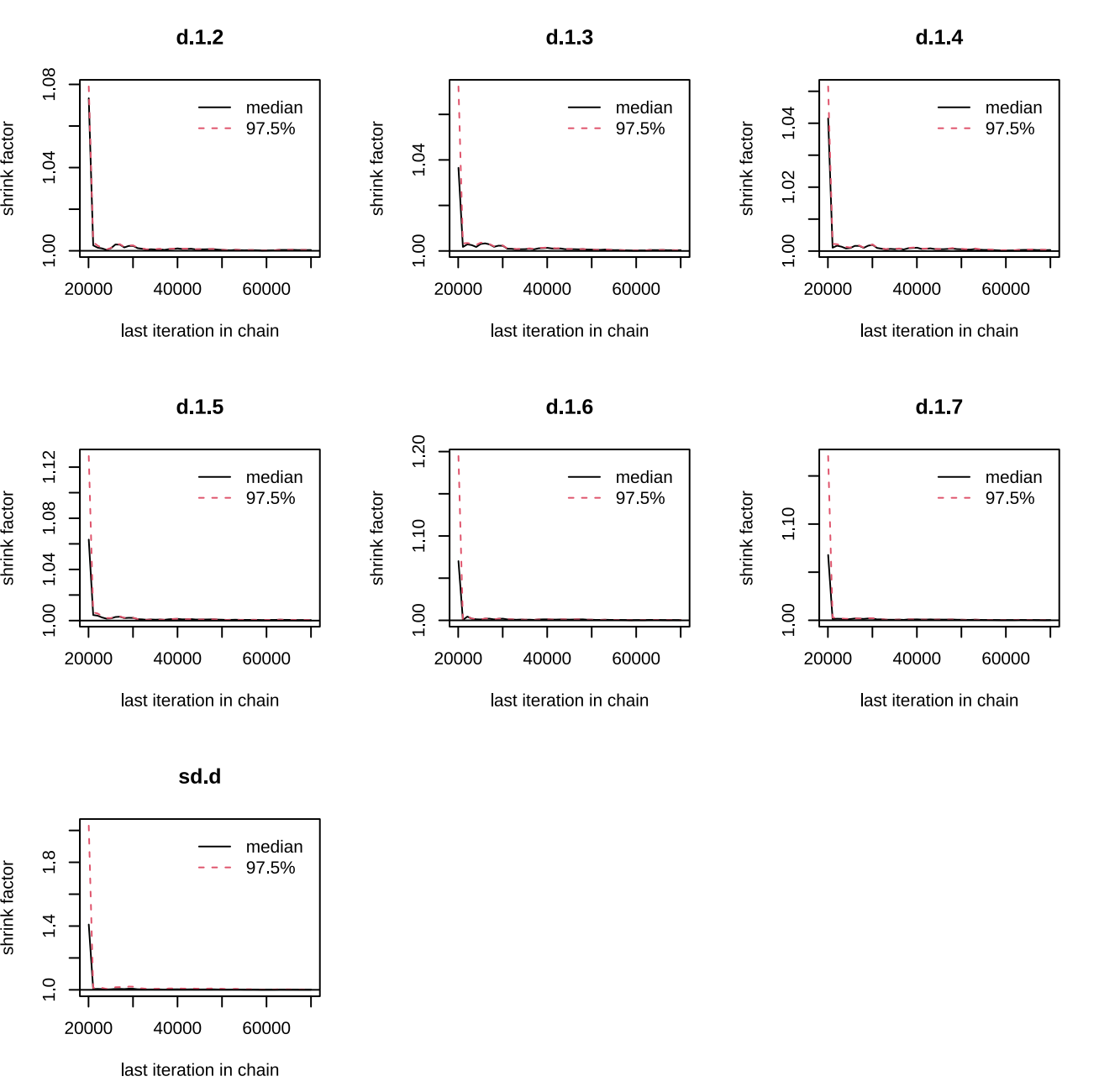


**Figure S2. diagnostic graph: BALP**


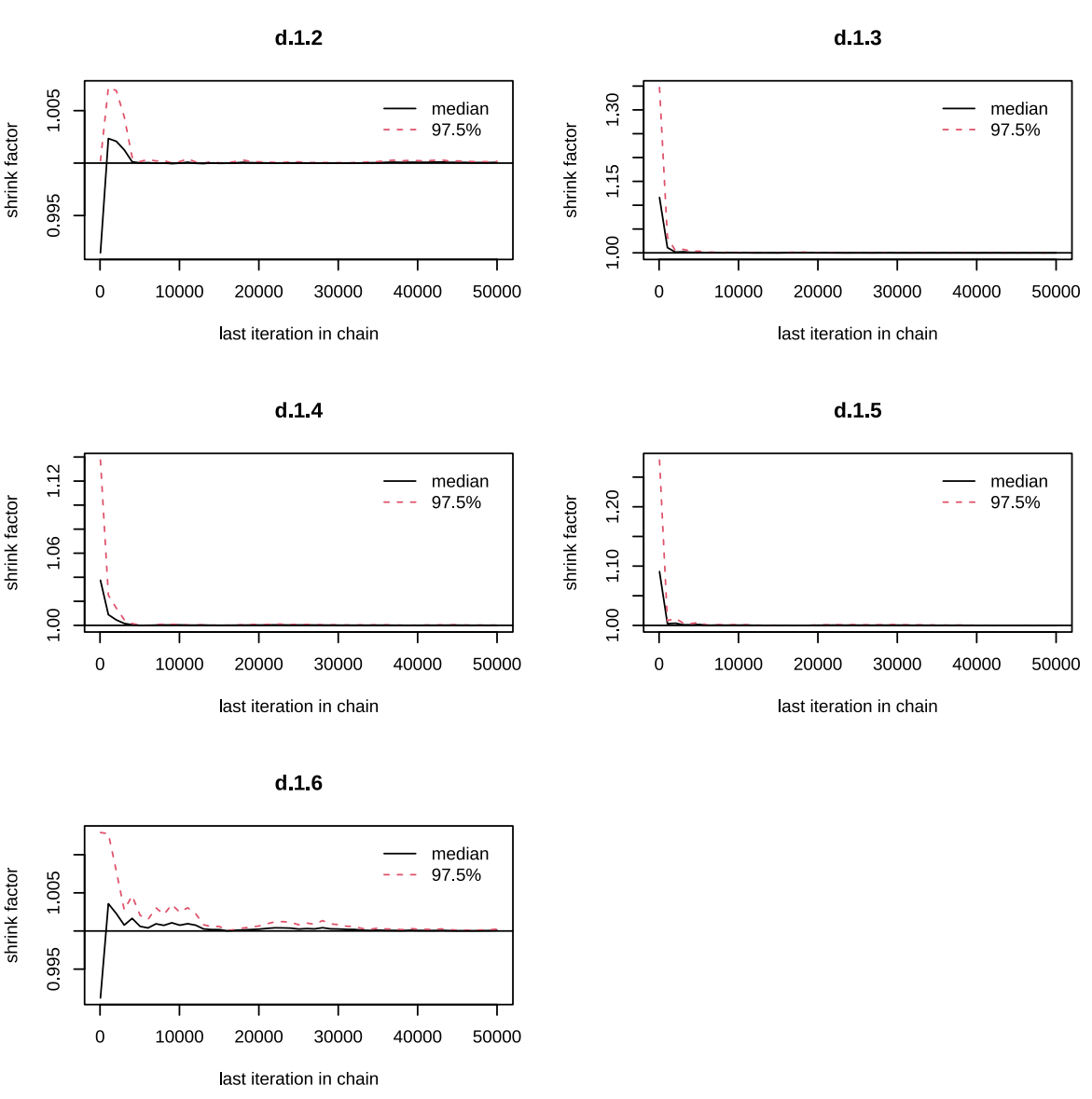


**Figure S3. diagnostic graph: Serum calcium**


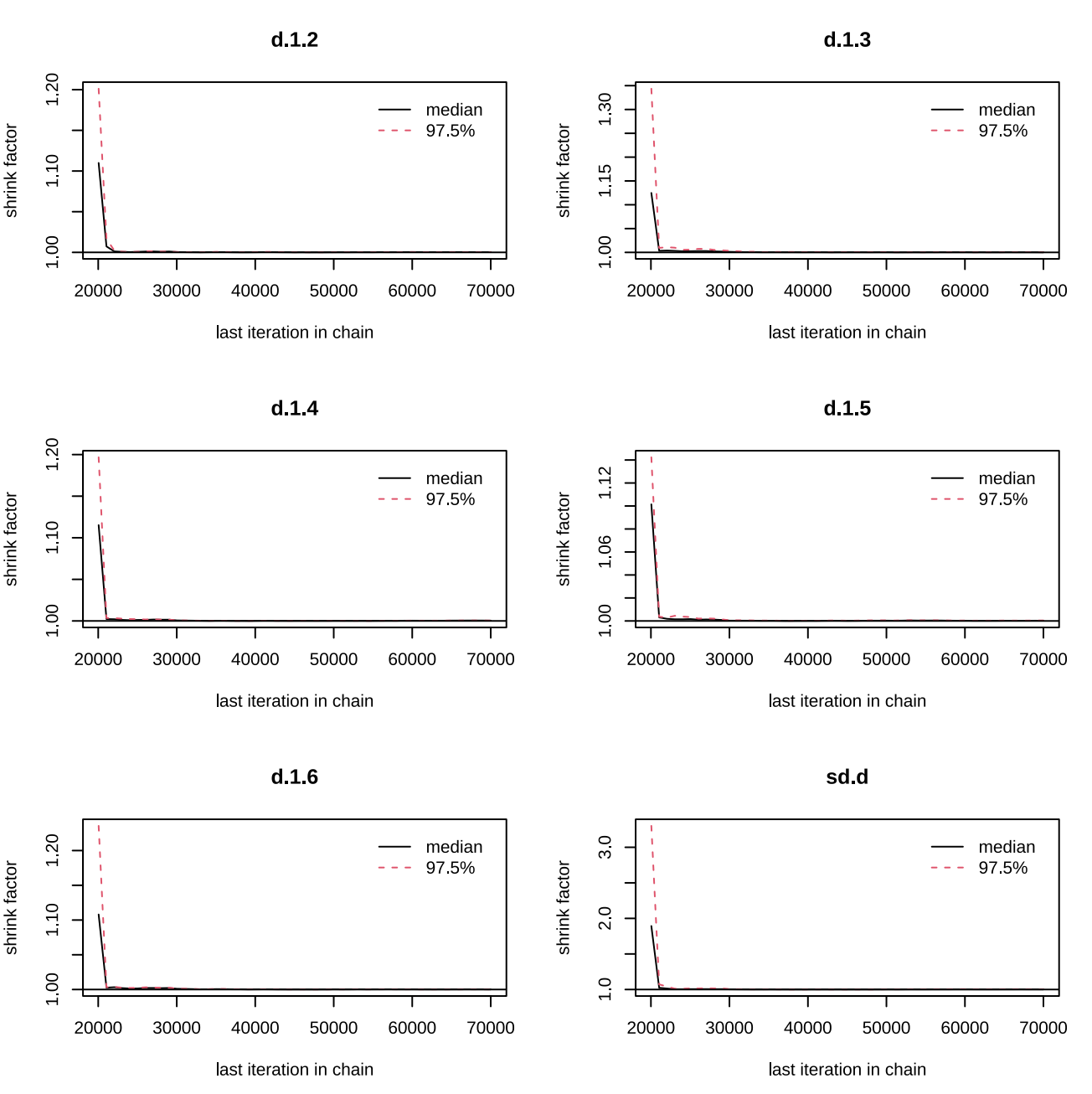


**Figure S4. diagnostic graph: Serum phosphorus**


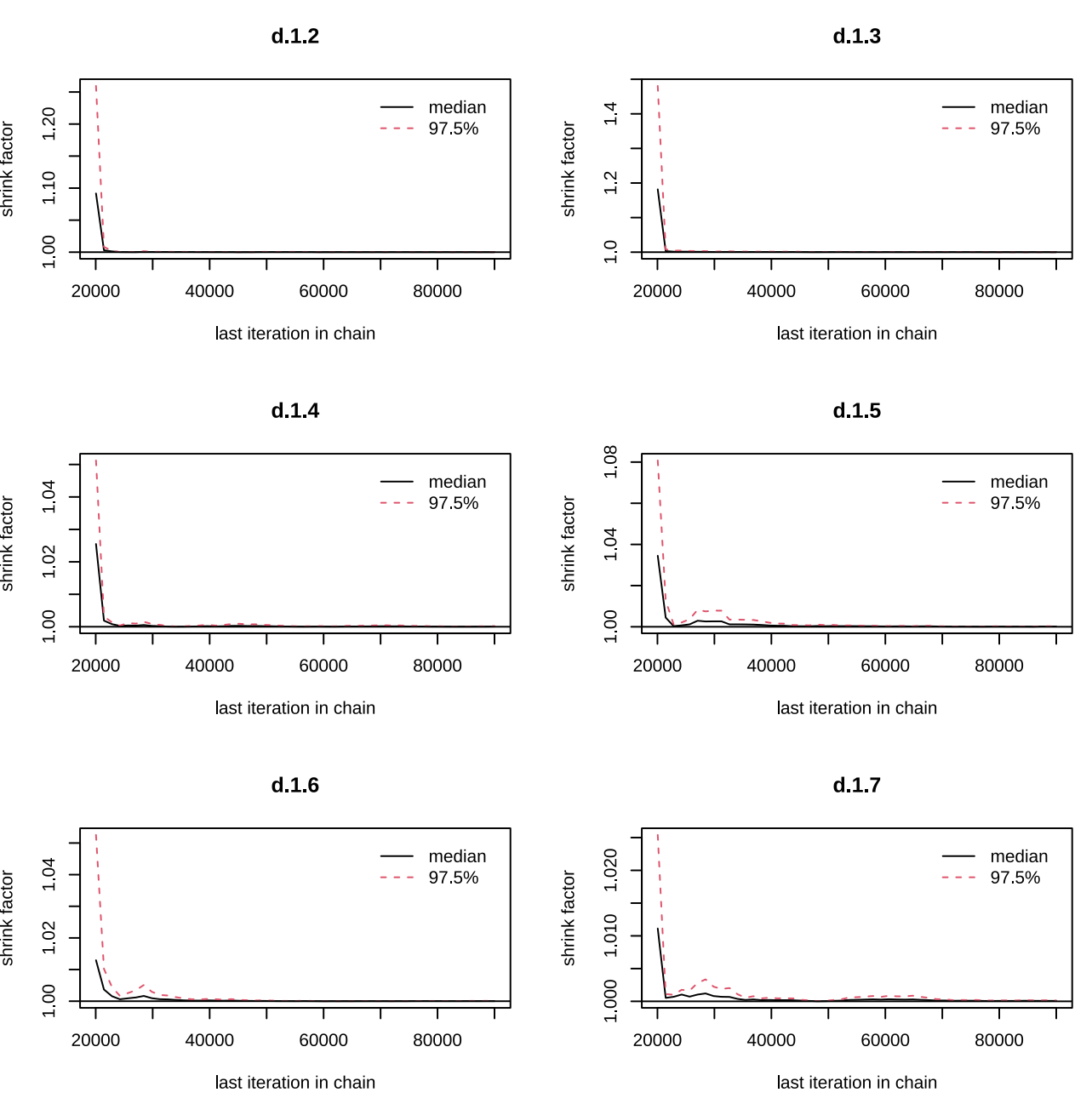


**Figure S5. diagnostic graph: Adverse actions**
