# Supplementary material for: Comprehensive evaluation of the efficacy and safety of different vitamin D combination regimens based on indirect comparisons for children with rickets: a network meta-analysis
Source: Front Nutr. 2026 Apr 8;13:1785775. doi: 10.3389/fnut.2026.1785775 (PMC13099536; doi:10.3389/fnut.2026.1785775)
Supplement: Supplementary file 3 [file Supplementary_file_3.docx]

**
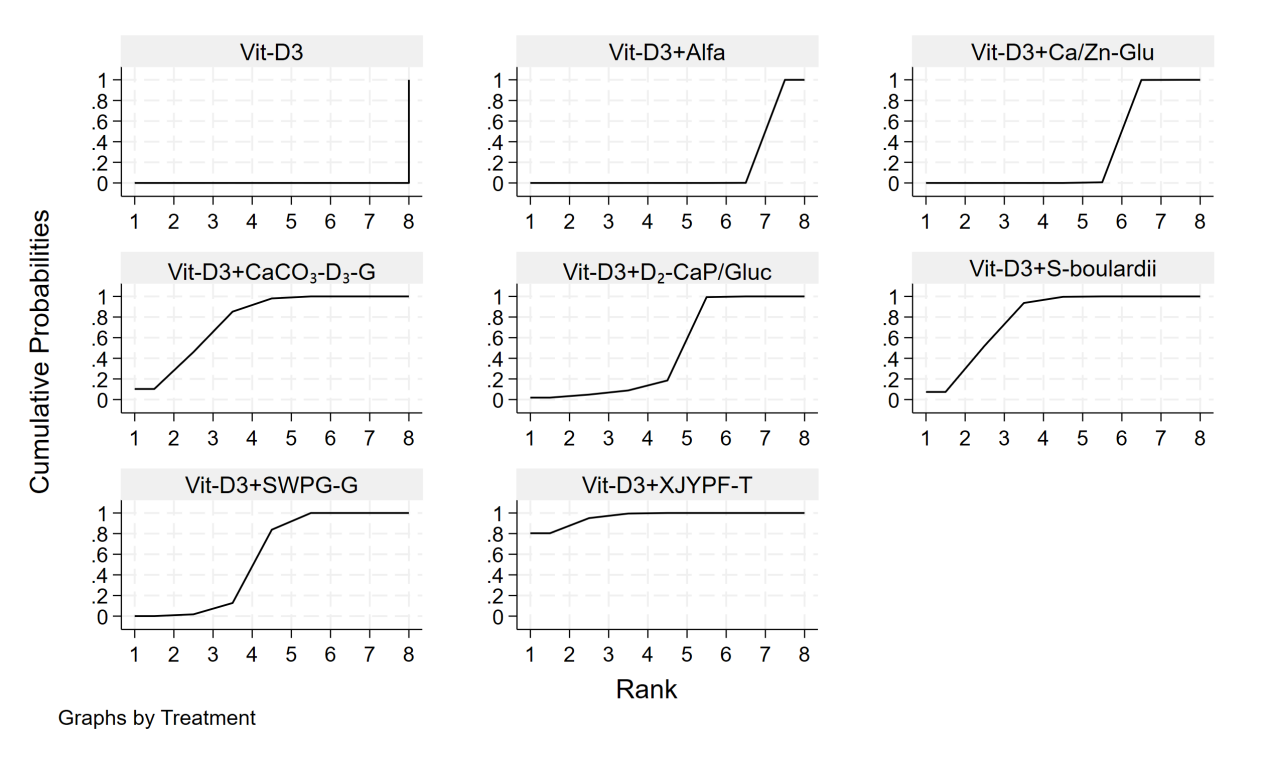
**

**Figure S1. SUCRA of vitamin D_3_ regimens on serum 25-(OH)D₃ concentration.**

**
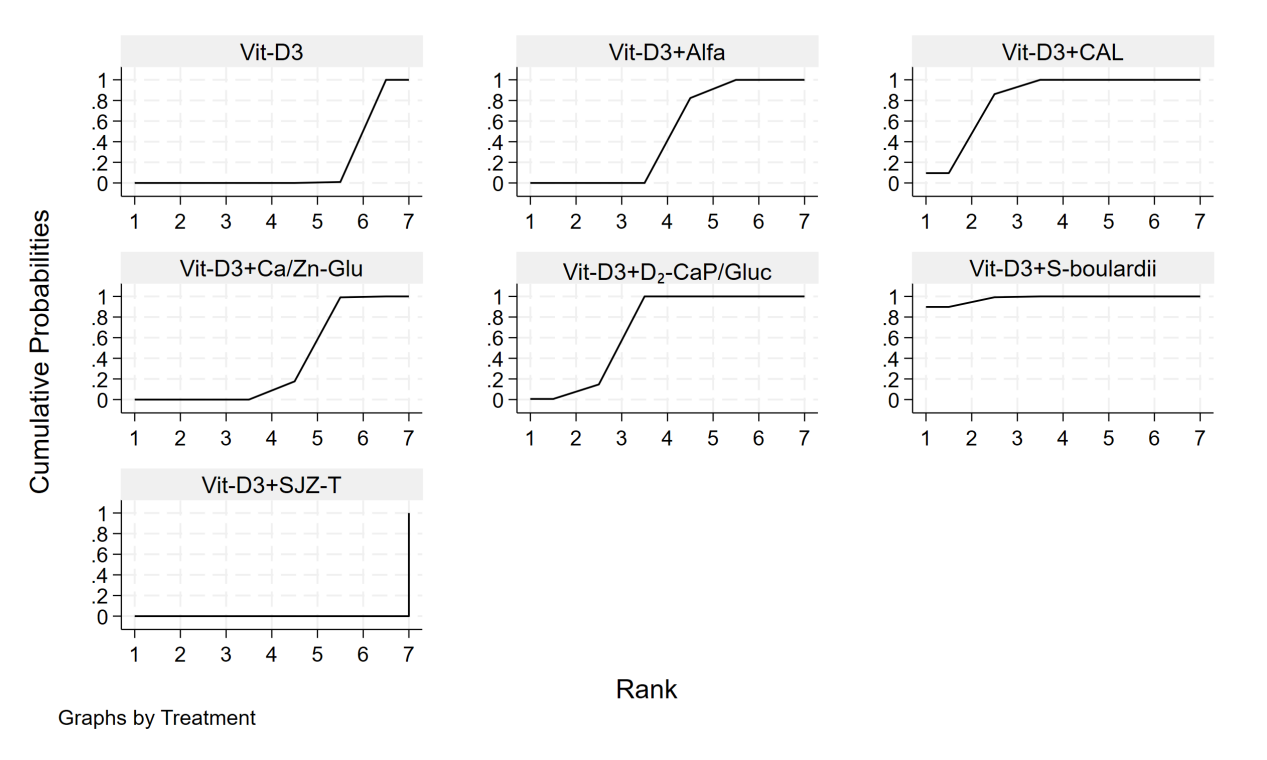
**

**Figure S2. SUCRA of vitamin D_3_ regimens on serum BALP concentration.**

**
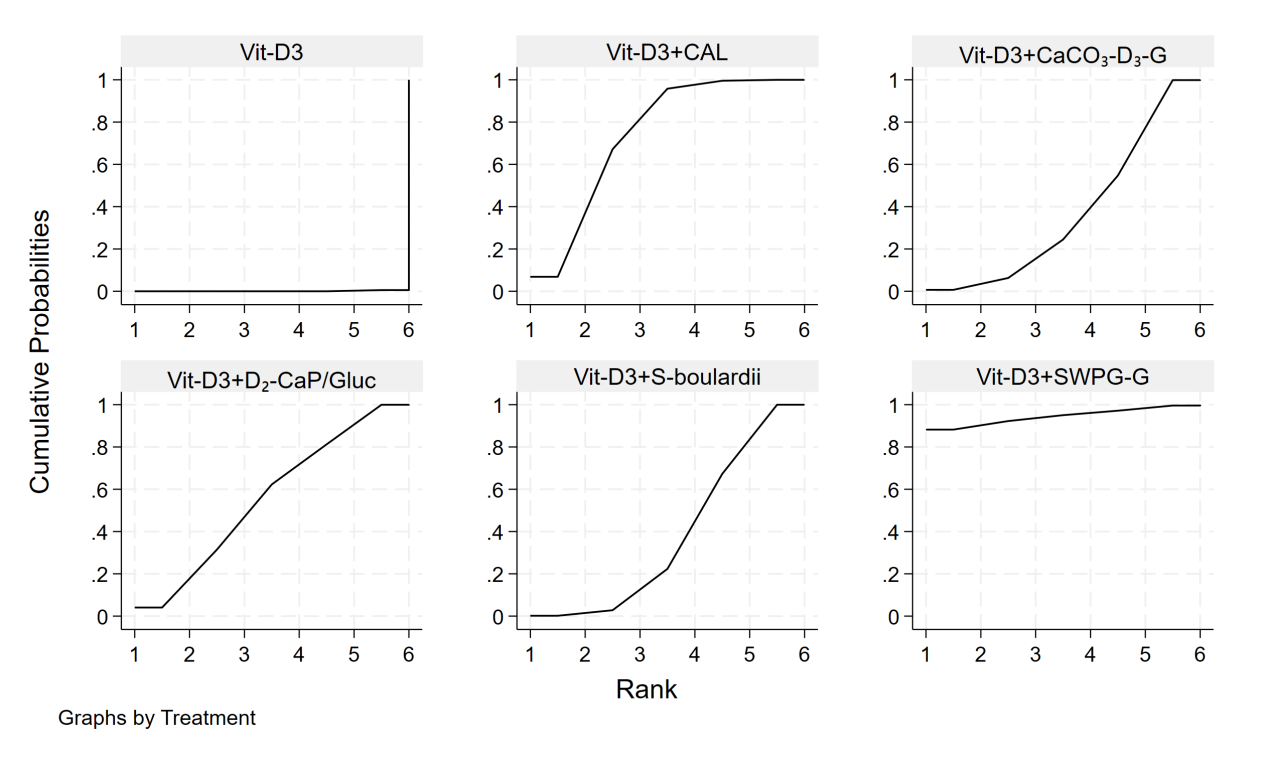
**

**Figure S3. SUCRA of vitamin D_3_ regimens on serum calcium concentration.**

**
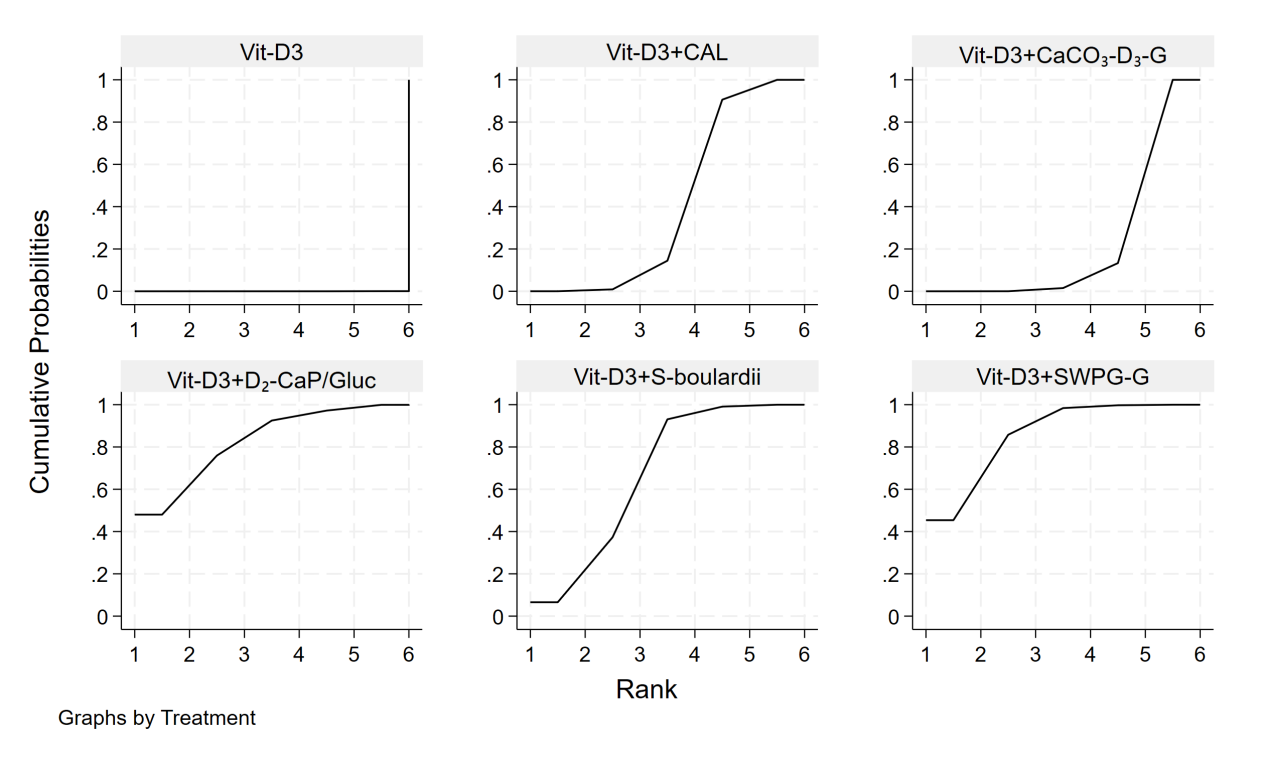
**

**Figure S4. SUCRA of vitamin D_3_ regimens on serum phosphate concentration.**

**
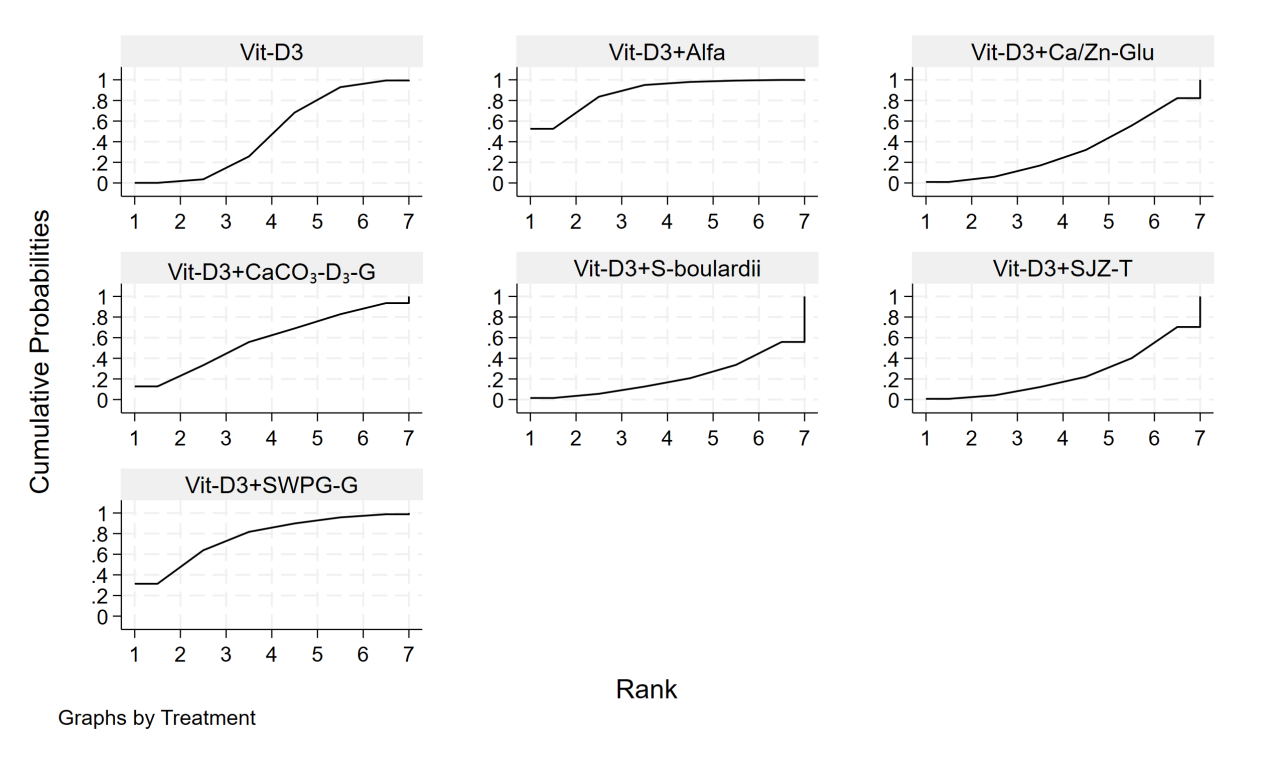
Figure S5. SUCRA of vitamin D₃ regimens on adverse events incidence.**
