# Supplementary material for: Comprehensive evaluation of the efficacy and safety of different vitamin D combination regimens based on indirect comparisons for children with rickets: a network meta-analysis
Source: Front Nutr. 2026 Apr 8;13:1785775. doi: 10.3389/fnut.2026.1785775 (PMC13099536; doi:10.3389/fnut.2026.1785775)
Supplement: Supplementary file 4 [file Supplementary_file_4.docx]

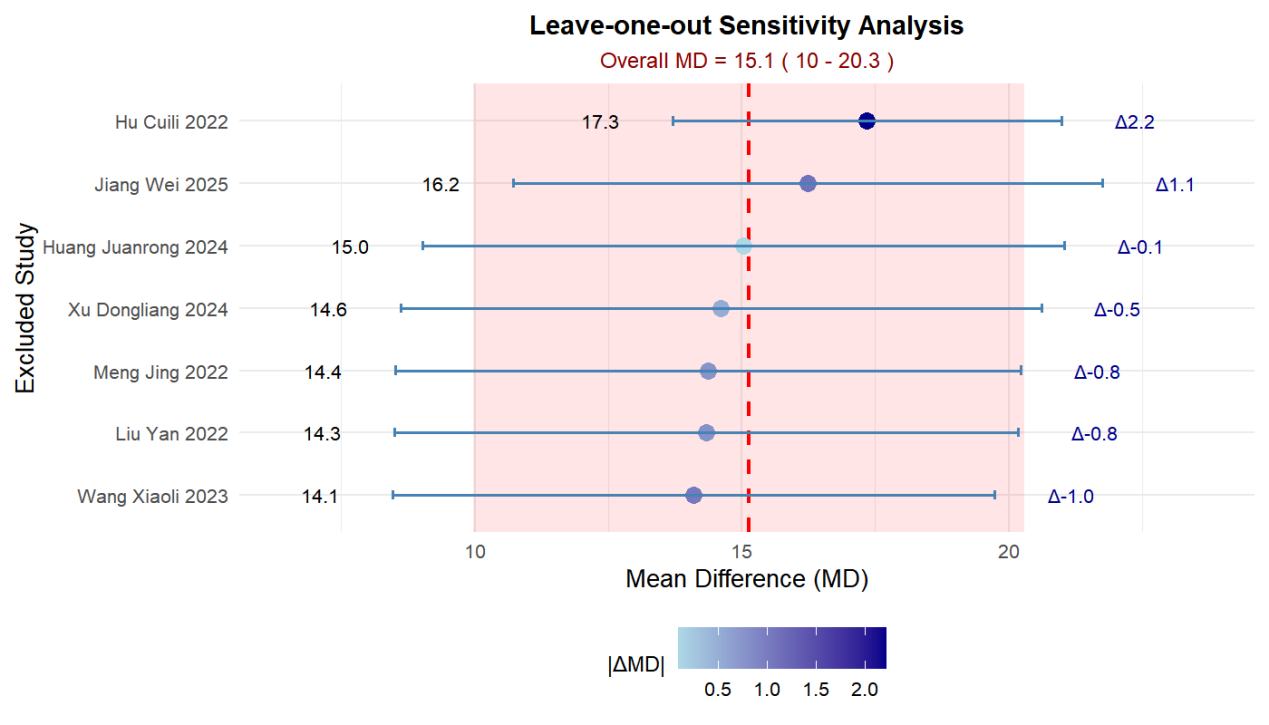


**Figure S1. Leave-one-out sensitivity analysis for serum 25-(OH)D₃ concentration.**

**
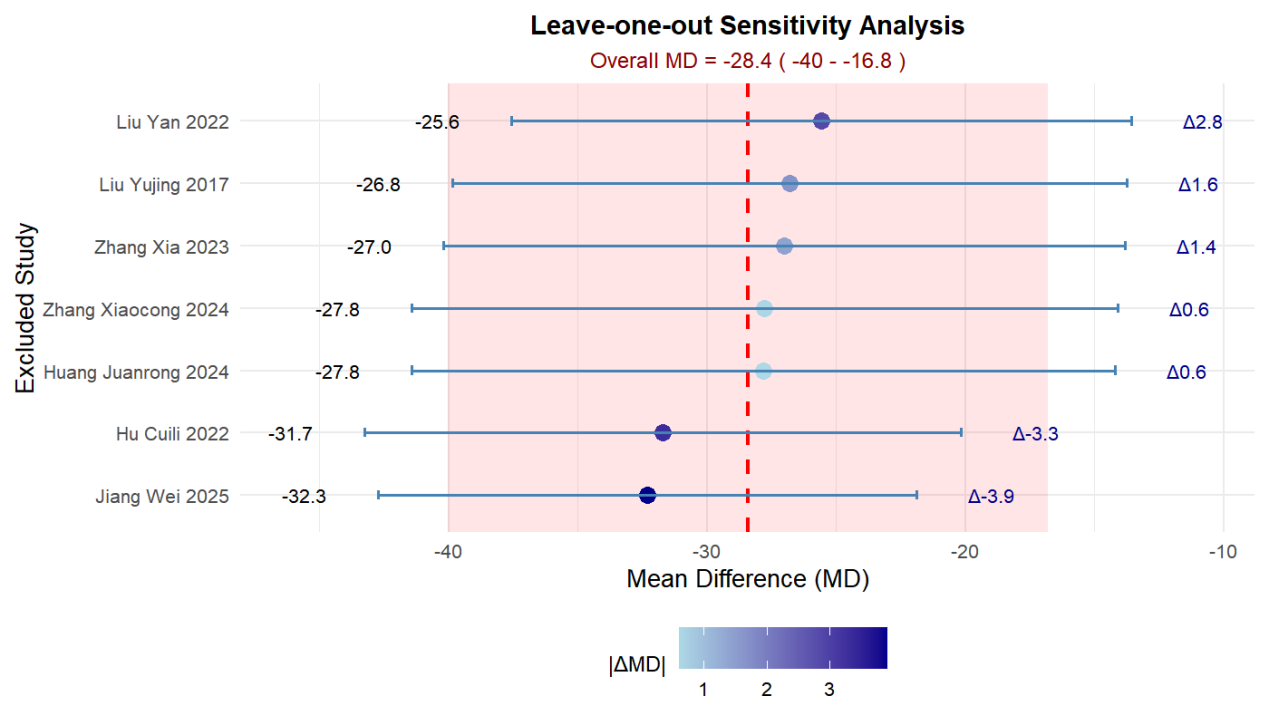
**

**Figure S2. Leave-one-out sensitivity analysis for serum BALP**

**
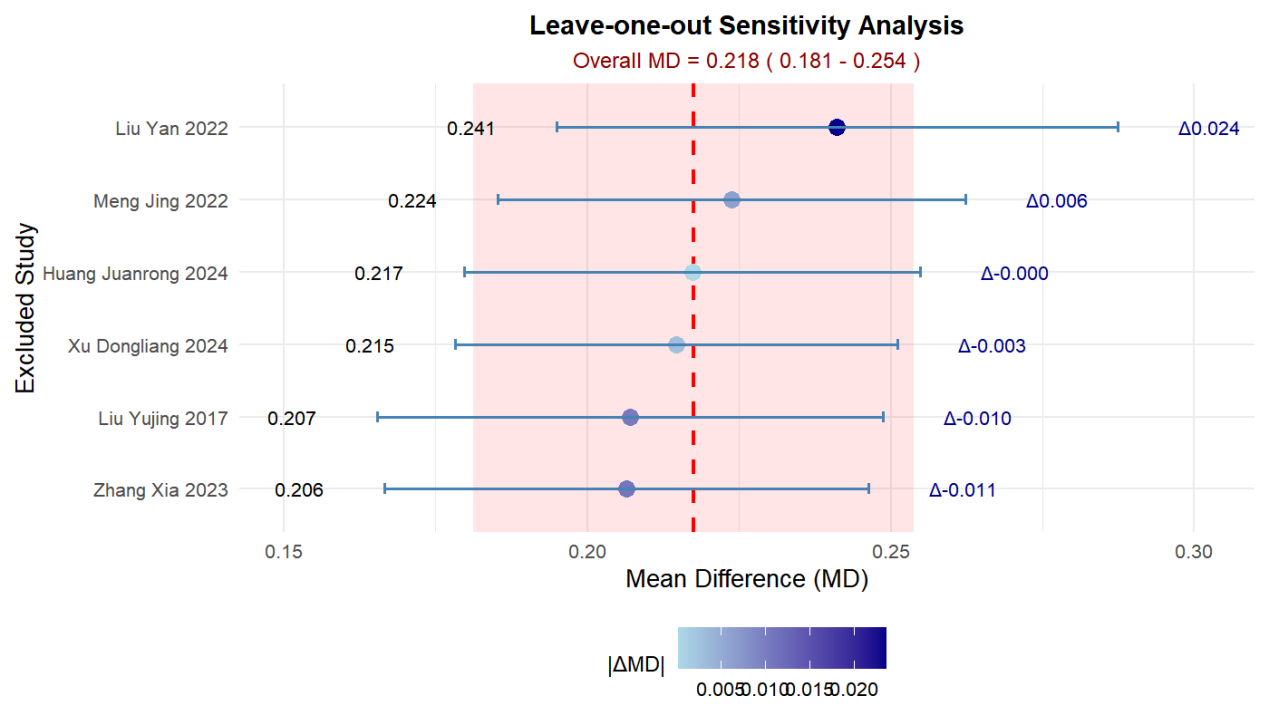
**

**Figure S3. Leave-one-out sensitivity analysis for serum calcium**

**
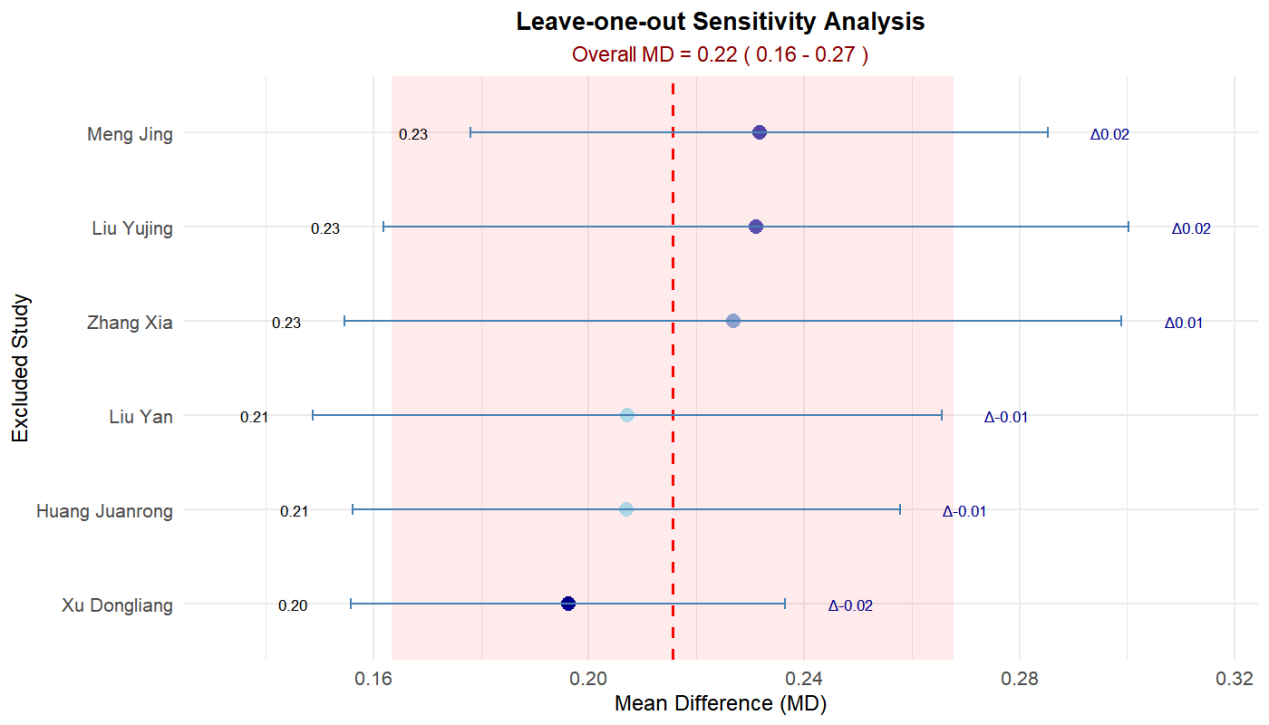
**

**Figure S4. Leave-one-out sensitivity analysis for serum phosphate**

**
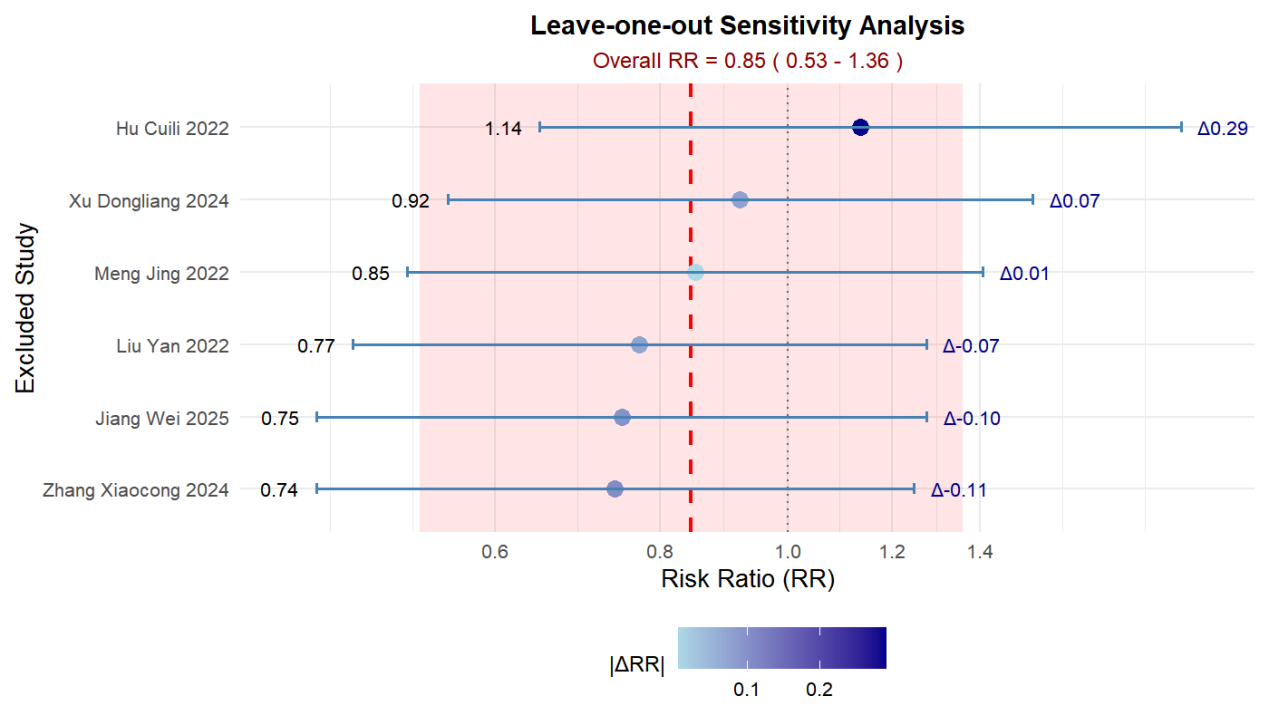
**

**Figure S5. Leave-one-out sensitivity analysis for Adverse actions**
